# The impact of planting times and cultivars on the excitation of innate-immunity response against populations and severity of plant parasitic nematodes in faba bean (*Vicia faba* L.) field

**DOI:** 10.1186/s12870-025-07148-8

**Published:** 2025-09-16

**Authors:** Rehab Y. Ghareeb, M. A. Gomaa, Hany S. A. Abd El- Latif, Essam E. Kandil

**Affiliations:** 1 Plant Protection and Biomolecular Diagnosis Department, Arid Lands Cultivation Research Institute, City of Scientific Research and Technological Applications (SRTA-City), New Borg El Arab, Alexandria, 21934 Egypt; 2 Plant Production Departments, Faculty of Agriculture (Saba Basha), Alexandria University, Alexandria, Egypt; 3 Plant Production Departments, Faculty of Agriculture (Saba Basha), Alexandria, Egypt

**Keywords:** *M. incognita*, RKN damaging, Real-time PCR, Host parasitic interaction

## Abstract

**Background:**

The most important food legume crop in Egypt and worldwide is the faba bean (*Vicia faba* L.), which has the potential to greatly improve agricultural sustainability by providing benefits such as higher grain yields, product diversification, and better nutritional value. It accounts for nearly 90% of the world's faba bean production. These long-season faba bean crops are very vulnerable to damage from root-knot nematodes (RKNs), the main threat to vegetable farming. This study aimed to assess how RKN damage affects faba bean production in Egypt's climate, considering different planting dates and cultivars, as well as the effects these factors have on the plants' natural immunity against *Meloidogyne incognita*. Two field experiments were carried out on sandy soil in Egypt’s North Delta during the 2021–2022 and 2022–2023 seasons to study how three faba bean cultivars (Nubaria 1, Giza 716, and Giza 843) respond to three planting dates under drip irrigation when infected with nematodes. A split-plot design with three replications was used. In both seasons, the three faba bean varieties (Nubaria 1, Giza 716, and Giza 843) were randomly assigned within the subplots, while the main plots included planting dates of October 1, October 15, and November 1.

**Results:**

Data on RKN population density and gall index were collected to evaluate the severity of *Meloidogyne incognita* and to detect other types of nematodes. The three examined cultivars exhibited mild to moderate *M. incognita* infections, alongside slight infections of *Xiphinema* sp. and *P. brachyurus* during the 2021–2022 seasons, with 80% of the roots galled. In contrast, during the 2022–2023 growing season, RKN damage was unaffected by the planting dates. However, delaying the planting date from October 15 to November 1 increased RKN population densities in the 2021–2022 season, as well as resulted in increases in plant height, total chlorophyll content, and shading percentage of faba bean plants. Additionally, on October 15, the plant immune system appeared to activate against plant-parasitic nematodes through the up-regulation of antioxidant enzymes and increased glucanase and chitinase (PPO, POD, and PR3).

**Conclusions:**

According to our findings, the tested faba bean cultivars Giza 716 and Giza 843 are highly vulnerable to RKN, which raises concerns about the potential harm RKN could cause to commercial faba bean production and underscores the necessity of managing RKN. However, it can be asserted that fewer nematodes will infect faba beans once Nubaria 1 is planted in Egypt in mid-October.

**Supplementary Information:**

The online version contains supplementary material available at 10.1186/s12870-025-07148-8.

## Introduction

Faba beans (*Vicia faba* L.) are cultivated across 58 countries over a vast area, making them the fourth most important pulse crop worldwide. They are versatile, used as grains, vegetables, animal feed, and green manure [1].They are versatile, used as grains, vegetables, animal feed, and green manure. The crop is extensively produced in countries like Egypt [2]. Faba beans are highly valued for their protein content, ranking seventh among legumes, and are significant for their role in nitrogen fixation, which enriches soil nitrogen from the atmosphere [3]. In industrialized nations, they are mainly used as animal feed for horses, poultry, and pigeons, whereas in developing countries, they are consumed by humans [4–6]. In Egypt, approximately 56,394 hectares, which make up about 85% of the total faba bean cultivated area, yielded around 10 ardeb fad-1 in northern regions. During 2018–2019, production reached roughly 135,345 tons, with a consumption of 420,000 tons (FAO, 2020) [7].

Despite their importance, low productivity and annual reductions in cultivated area suggest that local production cannot sufficiently meet demand. Poor yields are linked to factors such as low soil fertility, limited input use, weeds, sowing depth, inadequate soil moisture, poor sowing timing, plant density, and diseases. Faba beans thrive in diverse climates, from temperate to subtropical, and are vulnerable to pests, fungal diseases, viruses, and parasites, both native and introduced [8–10]. The three main varieties—spring, cool-temperate winter, and Mediterranean—are susceptible to pests and diseases at different growth stages. In autumn, when pest activity decreases, Mediterranean and winter varieties are sown, benefiting from relatively low temperatures until early spring. Although these varieties utilize available resources efficiently, they are almost always vulnerable to pests and diseases [11]. Conversely, spring-planted beans are exposed to pests and pathogens immediately upon sprouting, often facing a different set of threats. Root rot disease, caused by various soil-borne pathogens, is a major factor limiting plant growth and yield [12]. This disease, caused by multiple soil-borne pathogens affecting faba plants, is a significant concern. Notably, plant-parasitic nematodes, especially root-knot nematodes like *M. incognita*, pose substantial threats to faba beans and many other crops worldwide, damaging thousands of plant species [13]. Root rot disease, considered one of the main limiting factors for faba bean growth and yield, is caused by various soil-borne pathogens known to infect faba bean plants [13]. Among them, plant-parasitic nematodes trigger significant destruction to numerous crops worldwide. Root-knot nematodes (RKN) have become vital pests for vegetables and their host spectrum encompasses 3.000 plant species, particularly faba beans, heavily influenced by *M. incognita* root-knot nematodes [14, 15].

*Meloidogyne* spp., nematodes are plant-pathogenic nematodes that attack multiple crops. The species of root-knot nematodes mainly limited the yield of leguminous crops sown invaded soils [16].

Plant growth and yield are severely restricted by the economically damaging species of root-knot nematode *Meloidogyne incognita*, which causes an estimated $100 billion in losses annually [17, 18].

According to [19], plant root-knot nematodes are sedentary endoparasites that harm plant roots and decrease the root system's ability to absorb water and minerals. Rhizobial nodule growth is impacted when nematodes attack the roots of leguminous plants [20]. Thus, root nodule suppression impacted the plant's growth directly and indirectly.

Since the plant loses 40% of its photosynthesis, the rhizosphere has a relatively higher nutrient content than other soil components [21].

It is impossible to entirely eradicate nematodes; instead, the objective is to control their population and bring it down below harmful levels [21]. Conventional management practices include planting nematode-suppressive plants, applying pesticides, planting nematode-resistant crop varieties, rotating crops, flooding and land fallowing, adding soil amendments, and planting at the appropriate time [22]. Planting date is one of the most important limiting factors affecting plant growth [23]. It can have a major impact on the severity of diseases in faba beans; modifying the planting date to avoid periods of high moisture or extreme temperatures can help reduce the risk of infection and disease development [24].Therefore, crops planted earlier or later will be vulnerable to drought, harsh weather, pests, and disease attacks [25–27] found that late sowing increased the severity of insect and disease attacks; it also decreased the length of the green pod, the number of seeds per pod, the number of days until flowering, and the seed yield. It was discovered that later sowing dates decreased disease invasion and infection, even though the optimal sowing date produced the highest seed yield [28–30]. Therefore, to prevent further infection by pathogens, the inducers of plant resistance must activate the innate defence mechanisms. Plant species differ in the complexity and effectiveness of their defence mechanisms against attacks by different pathogens [31]. The system's defence mechanisms include the generation of antimicrobial molecules on the cell walls, such as phytoanticipins, cutins, wax, and rigid lignin. These are typically regarded as the first line of defence to stop further pathogen invasion [32].

To combat these pathogens, they must have used a different defence strategy, as many of them have a first line of defence. The pathogen-induced defence response is one such defence mechanism that consists of a hypersensitive reaction, reactive oxygen species (ROS) production, cell wall cross-linking, the synthesis of antimicrobial molecules such as phytoalexins, and ultimately the production of PR proteins, [33]. PR proteins are among them; they are essential components of SAR, an inducible plant immune response that stops additional infections from uninfected parts of the host organism [34].

Our study aims to explore the immediate effects of different sowing dates with various cultivars on nematode severity, infection, and growth parameters of faba bean. Additionally, we aim to provide recommendations for faba bean health and production under nematode-infected soil conditions in the North Delta of Egypt to improve understanding of how different sowing dates and genotypes influence root-knot nematode invasion responses.

## Materials and methods

### Plant materials

Three faba bean cultivars (Nubaria 1, Giza 716, and Giza 843) were obtained from Nubaria Station Research, Agricultural Research Center (ARC), Giza, Egypt, and used in this study.

### Experiment site and time

Field experiments were conducted in the Nubaria region, El-Bahira, Egypt (30°40′N 30°04′Eⁿ/30.667°N 30.067°Eⁿ/30.667; 30.067). The soil at the study site is classified as sandy clay loam and has a low content of organic matter, very high calcium carbonate, and is non-saline. The available amounts of macro-elements were moderate for nitrogen and low for phosphorus and potassium. Regarding available concentrations of micronutrients, Fe, Cu, and Mn were at medium levels in the soil, while Zn and B were in low amounts. At the beginning of this study. The naturally occurring infestation of the soil with *Meloidogyne incognita*, which is a common RKN species at the field study location, under organic conditions using a randomized full block design with three replications and without the use of any chemical inputs during the growing seasons of 2021/2022 and 2022/2023, two field experiments were carried out to investigate the effects of planting dates on particular faba bean cultivars and their interaction with productivity and plant pathogen nematodes. Plots in the trial were 3 m long, with 6 rows and 45 cm between rows and 10 cm between rows. Blocks and plots were separated by 1 m and 0.5 m, respectively. Following hand sowing, sprinkler irrigation was used to meet the plants'water needs until they emerged and the first weeds were removed. A drip irrigation system was then set up, consisting of one drip line per row. The three faba bean cultivars (Nubaria 1, Giza 716, and Giza 843) occupied the subplots, while the main plots were planted on the first of October, mid-October, and the first of November. Dates for planting (main plot): first of October, mid-October, and first of November. Cultivars of faba bean (sub-plot): Nubaria 1, Giza 716, and Giza 843. Nubaria Station Research, Agricultural Research Center (ARC), Giza, Egypt, is the source of the three faba bean cultivars. Following plowing once, the field was fertilized with phosphorus fertilizer, which was applied as a single calcium superphosphate (15.5 P₂O₅) at a rate of 480 kg/ha and potassium sulphate (48 percent K₂O) at a rate of 120 kg/ha before planting with soil preparation. However, with the initial irrigation, a nitrogen fertilizer (120 kg N/ha) was applied as urea fertilizer (46% N); before beginning the experiment, representative soil samples were extracted from the experimental soil for analysis [35].

### Climate in the research region

The average temperature recorded in 2022 was higher than the long-term average during the trial vegetation period (October–November), while the monthly average relative humidity values were somewhat lower than the long-term average. Monthly average temperatures for the second year (2023) were found to be between 24°C and 32°C. The average relative humidity for each month, however, was found to be between 65.5 and 68%.

### Growth parameter

The average plant height (cm) of ten plants at three different growth stages—100, 114, and 128 days after sowing (DAS)—was measured from the soil's surface to the top of the plant.

### The content of total chlorophyll

The instrument used was a Minolta Co., Ltd. SP AD-502, produced in Japan. From three weeks after transplanting (WAT), chlorophyll content was measured in the five fully expanded leaves from the top of the faba bean; each leaf's SPAD value was recorded per plant, based on its light transmission in two wavelength ranges affected by *chlorophyll* absorption.

### Percentage shedding

It was calculated using the following equation: Shedding percentage = 100—Pod setting percentage.

### Assessment of RKN damage severity

Gall/root inspections on ten roots/plot were done according to [36] as follows:

1. a 0–10 score where 0 indicated no visible galling roots
2. 10 displayed 100% galled with no visible fibrous roots

250-g soil samples were gathered from the planting holes around roots for RGI examination using the Baermann pan method with slight modifications and incubated for two days at 27 ± 2°C to detect and count the nematode population [37–39]. After 120 days of faba bean planting, root systems were gently freed and washed in tap water to remove them from the soil. After cutting root systems into 2.5 cm segments, subsamples were randomly chosen for nematode extraction and either ground in 0.5% NaOCl or stirred in 60 ml of water [40]. In a Warring blender equipped with a 500 ml pulverizing container, root tissue was ground as quickly as possible. We poured the contents of the pulverizing container (ground treatments) or beaker (stirred treatments) onto sieves with 75- and 25-μm pores. The second juvenile stage of nematodes (J2s) and eggs gathered on the sieve at 25-μm-pore were counted; nematode-infected roots were collected and submerged in a perfect solution of 0.015% phloxine B/15 min for the egg masses staining [41] to count them as well; the nematode eggs were then removed from the faba bean roots using the [31] method with slightly modified. Evaluating the nematode's ability to infect, the total number of eggs was considered because it indicates the number of J2s that were able to penetrate the root tissue, infect it, and develop into females that lay eggs. The number of egg masses was also examined and counted, [42]. Lastly, adult nematode females were extracted from galled roots in order to identify the species [32].

### Molecular studies

#### The mode of action of the faba beans'response at different planting times

##### RNA extraction of faba bean leaf

RNA isolation from faba bean plants that were sown as controls (the first two leaves after sowing) on October 1, 2021, October 15, 2021, and November 1, 2021, following the TRIzol reagent protocol. Using a pre-cooled pestle and mortar, the tissues of the faba bean plants were ground into a fine powder in liquid nitrogen before being placed individually in a 50 ml plastic Eppendorf centrifuge tube. One gram of samples was mixed thoroughly with one millilitre of extraction buffer [TRIzol reagent: 38% phenol (USB Cooperation, Cleveland, Ohio, USA)] [43] that had been adjusted to pH 4.0 using Tris–HCl buffer, 0.8 M guanidine thiocyanate, 0.4 M ammonium thiocyanate, 0.1 M sodium acetate (pH 5.0), and 5% glycerol. The samples were incubated at −20 degrees for 15 min; following incubation, tubes were vortexes for 15 s after adding 0.2 mL of chloroform to each mL of buffer extraction. After five minutes of room temperature incubation, the tubes were centrifuged for fifteen minutes at 12,000 rpm and 4°C. After carefully transferring the aqueous layer into a sterile Eppendorf centrifuge tube, 0.5 ml of isopropanol was added for every 1 ml of extraction buffer. Tubes were covered and mixed by gentle inversion, and then sit at room temperature for 10 min, tubes were centrifuged (12,000 rpm; 4°C; 20 min) and the supernatant was discarded. Pellet washes 75% ice-cold ethanol and centrifuged (10,000 rpm; 4°C; 20 min) and the supernatant was discarded, dry pellet for 10 min. RNA pellet was dissolved in 50 µl RNase-free water. RNA purity was additionally verified using an ethidium bromide-stained 1% w/v agarose gel, and the total RNA concentration was measured by the absorbance at 260 and 280 nm via a Spectrostar Nano (BMG LABTECH GmbH, Germany). Samples were stored at −80°C until further use.

##### Complementary DNA (cDNA) synthesis

Following the standard protocol reverse transcriptase (M-MLV, RT, Fermentas, USA) and buffer (5X) [50 mM Tris–HCl (pH 8.3 at 25 OC), 250 mM KCL, 20 mM MgCl_2_, and 50 mM DTT] were used to synthesize cDNA for the first strand. Random hexamer primers (Promega, USA) were also present [44] with slight modification [45]. Three microliters of RNA were added to 10 µl (5x) RT-Buffer, 2.5 mM dNTPs, 5 µl of primer, 0.3 µl (20 u/µl) of RT-enzyme, and 2.2 µl H2O. To perform the RT-PCR amplification, the mixture was incubated in a thermal cycler (Eppendorf, Germany) for 60 min at 37°C and then for 10 min at 70°C (to inactivate the enzyme). It was then stored at 4°C until it was required.

##### Differential Display-PCR (DD-PCR)

Genes that were up-and-down-regulated were detected using the differential display PCR (DD-PCR) technique. First, the PCR amplification reaction will be incubated at 94°C for 5 min with cDNA, PTC-100 TM (programmable Thermal controlled MJ Research Ch, INC). This will be followed by 40 cycles of 94°C for 30 s, 42°C for 1 min, and 72°C for 1 min. A five-minute extension period will be at 72°C. Using 0.5 × TBE as a running buffer, the resultant PCR products will be examined in 1.5% agarose gels by electrophoresis apparatus with power supply 500–600 V., E 865 (Biometra, USA)., which was carried out at 80 V. The gel was then stained with 0.5 µg/cm3 (w/v) ethidium bromide solutions and deionized water. Lastly, a gel documentation system (Alpha-chem. Imager, USA), will is used to photograph the DNA fragments and visualize them under UV light transilluminator (Cola-Parmer, USA). Additionally, PCR amplification was performed using two decamer oligonucleotides following the [46] method. Three primers were reconstituted in a stock solution of Millie-Q water at concentrations of 100 pmol/µl. Each primer's working solution was made with 10 pmol/µl of PCR reaction. A 25 µl reaction mixture comprising 12.5 µl Master mix, 2 µl of each primer (40 pmol/l) (Table 1), 3 µl cDNA, and 5.5 µl dH2O was used for the amplification reactions.

**Table 1:** Names and sequences of primers are used

Primers name	Nucleotide sequence 3 5	Ref
ITS-1	TCC GTA GGT GAA CCT GCG G	[47]
ITS-4	TCC TCC GCT TAT TGA TAT GC	[47]
PR3 R	GCG GAT CCC AAC GCA CTG CAA CCG ATT AT	[48]
PR- 1 (F)	TTC TTC CCT CGA AAG CTC AA	[48]
Polyphenol oxidase (PPO) F	C A T G C T C T T A T G A G G C G T A	[49]
Polyphenol oxidase (PPO) R	CCATCTATGGAACGGGAAGA	[49]
Chitinase (Chi) F	AAT GAT GCC GCT TGT CCT G	[50]
Chitinase (Chi) R	TCC ATA ACC CGG TAA TCT CCC	[50]

In a thermal cycler, the amplification was carried out for 40 cycles. Each cycle included one minute of denaturation at 95°C, followed by a post-extension step lasting ten minutes at 72°C following the conclusion of the previous cycle and an initial delay of four minutes at 95°C at the start of the first cycle [51]. PCR products were separated using 1.5% (w/v) agarose dissolved in 0.5 X TBE buffer on agarose gel electrophoresis. DNA molecular markers were used to estimate each band's size. By using the gel documentation system, the gel was finally photographed.

##### Quantitative real-time PCR (Q-PCR)

Comparative analysis of defence’s gene expression levels using quantitative real-time PCR (Q-PCR). Faba bean leaf samples were collected at different planting intervals on 1 st October 2021, 15th October 2021, and 1 st November 2021 and controlled. TRIzol Reagent (Invitrogen, USA) was used to extract the total RNA of faba bean samples following [43] and the user's manual. In this experiment, three gene-specific primers were used (Table 2). A total of 25 μl was used, which included 2 μl of cDNA as template, 1 μl of 25 pM/μl forward primer, 1 μl of 25 pM/μl reverse primer, 12.5 μl of cyber green, and 6.5 μl of RNase-free water. Before being loaded into the rotor's wells, the samples were spun. Initial denaturation at 95°C for 10 min, 40 cycles of 95°C for 15 s, annealing at 60°C for 30 s, and extension at 72°C for 30 s comprised the real-time PCR program. The extension step involves the acquisition of data. The Rotor-Gene 6000 system (Qiagen, USA) was utilized to carry out this reaction. According to [35, 38], qRT-PCR analysis of related genes was performed. Total RNA was measured using the ITS gene as a reference. Following the completion of the PCR program, the data were examined using [52] comparative Ct (2ʾΔΔct) method.

**Table 2:** The *M. incognita*, gall counts, egg masses per plant and Number of J2s/250 g of soil infected cultivars of faba beans (*Vicia faba* L.) with defferent plantind dates

Cultivars	Planting date	No. of gall/plant	No. of egg masses/plant	Number of J2s/250 g of soil
Control	-	825^a^	1520	320^a^
Giza 716	1^st^ October	140^d^	80^de^	80^a^
	15^th^ October	110^cd^	69^e^	73^cd^
	1^st^ November	718^b^	1100^bc^	288^b^
Giza 843	1^st^ October	40^ef^	20^f^	80^cd^
	15^th^ October	31^ef^	10^g^	59^e^
	1^st^ November	0	20^f^	60^e^
Nubaria 1	1^st^ October	5^g^	10^g^	23^f^
	15^th^ October	0	0	18^f^
	1^st^ November	43^ef^	20^f^	77^cd^

Data are means of 10 replicates; means with the same letter(s) in each column are not significantly different at *P* ≤ 0.05

### Analysis of molecular data experiments

The Rotor-Gene-6000 Series software was used to perform a comparative quantitative analysis of the samples. In order to determine gene expressions using ITS (reference gene) and other defense genes, Delta Delta Threshold cycle (ΔΔCq) expression values were computed for RNA samples of each faba bean treatment (Control and 1 October 2021, 15 October 2021, and 1 November 2021); Δ C q = C q – Housekeeping gene; ΔΔ C q = C q – Control; ΔΔ C q expression = 2 (-ΔΔCq). The equations show the mathematical model of the relative expression ratio for the real-time PCR. The ratio of the target gene is expressed in the faba bean plant sample control in contrast to the housekeeping gene. Rotor-Gene-6000 version 1.7 was used to statistically evaluate, interpret, and analyze the data.

### Statistical analysis

All data obtained were exposed to analysis of variance [53]. All statistical analyses were performed via the variation analysis technique utilizing the CoStat computer software package. The treatment means were compared using the least significant differences test (LSD) at the 5% probability using the CoStat 6.311 [54] statistical program. The morphological, biochemical, and phytochemical data were analyzed using two-way ANOVA along with Tukey’s multiple comparison test, conducted with GraphPad Prism 9 (GraphPad Software, Inc., San Diego, CA, USA). The results are presented as means ± SDs, with P values ≤ 0.05 indicating significant differences. The UPGAMA cluster, identified by the PAST software [108], employed principal component analysis (PCA), and the color Pearson correlation effectively depicted the similarity levels among different soil parameters based on the Dice coefficient, using the R Studio interface and R software [R1] [55, 56].

## Results

**Fig. 1:**
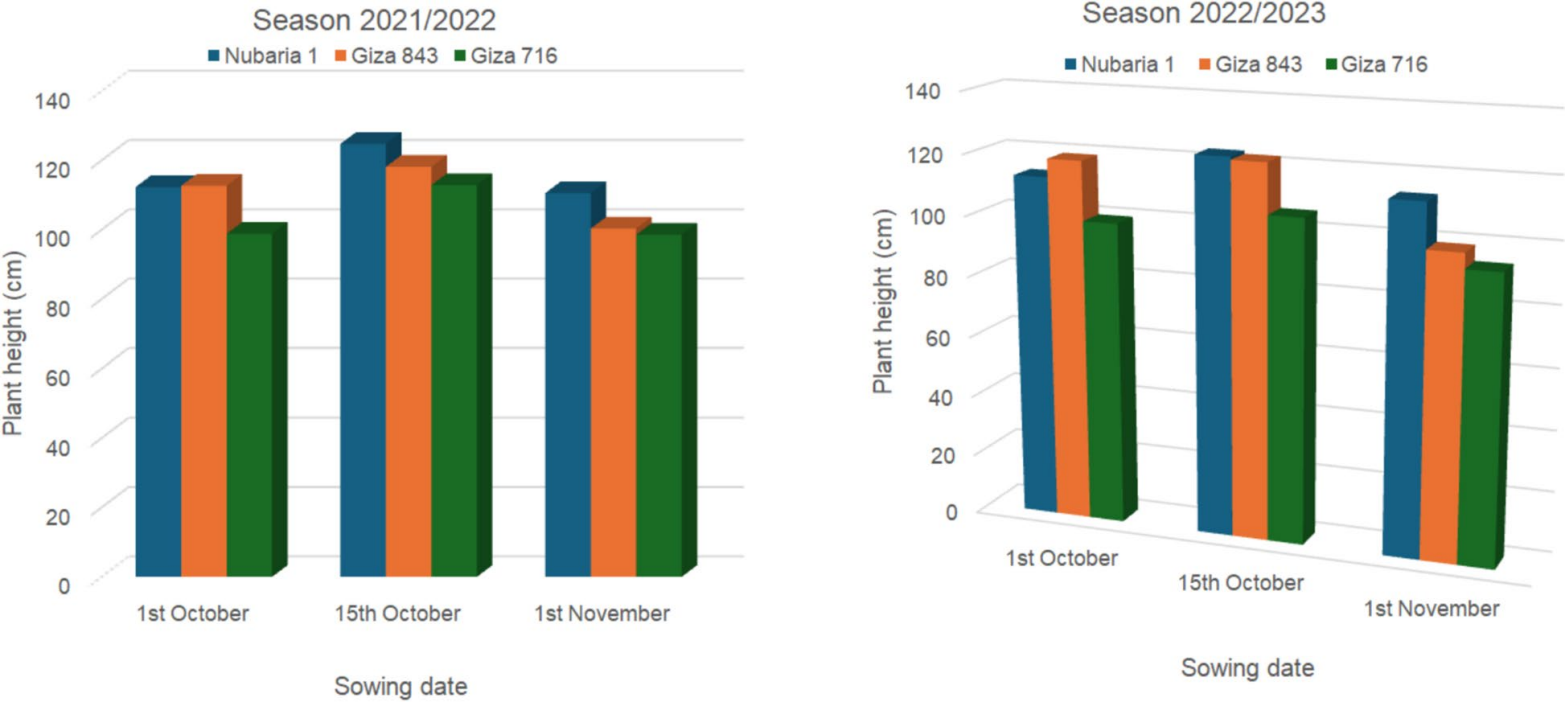
**a** Plant hieght (%) of the three faba bean varieties as affected by planting dates and their interaction in 2021/2022 season; **b** in 2022/2023 season

In the 2021–2022 and 2022–2023 seasons, Fig. 1a & b displays three faba bean cultivars that responded to planting dates and the dates with plant height interaction. Regarding the planting date affecting plant height, the second date, mid-October, had the highest values, followed by the first date, October 1st. In contrast, over the two seasons, there was no discernible difference between the first and second dates. This might be due to the planting date resulting in an extended period of vegetative growth, and this resulted from the improvement of several agronomic characters.

### Contents

**Fig. 2:**
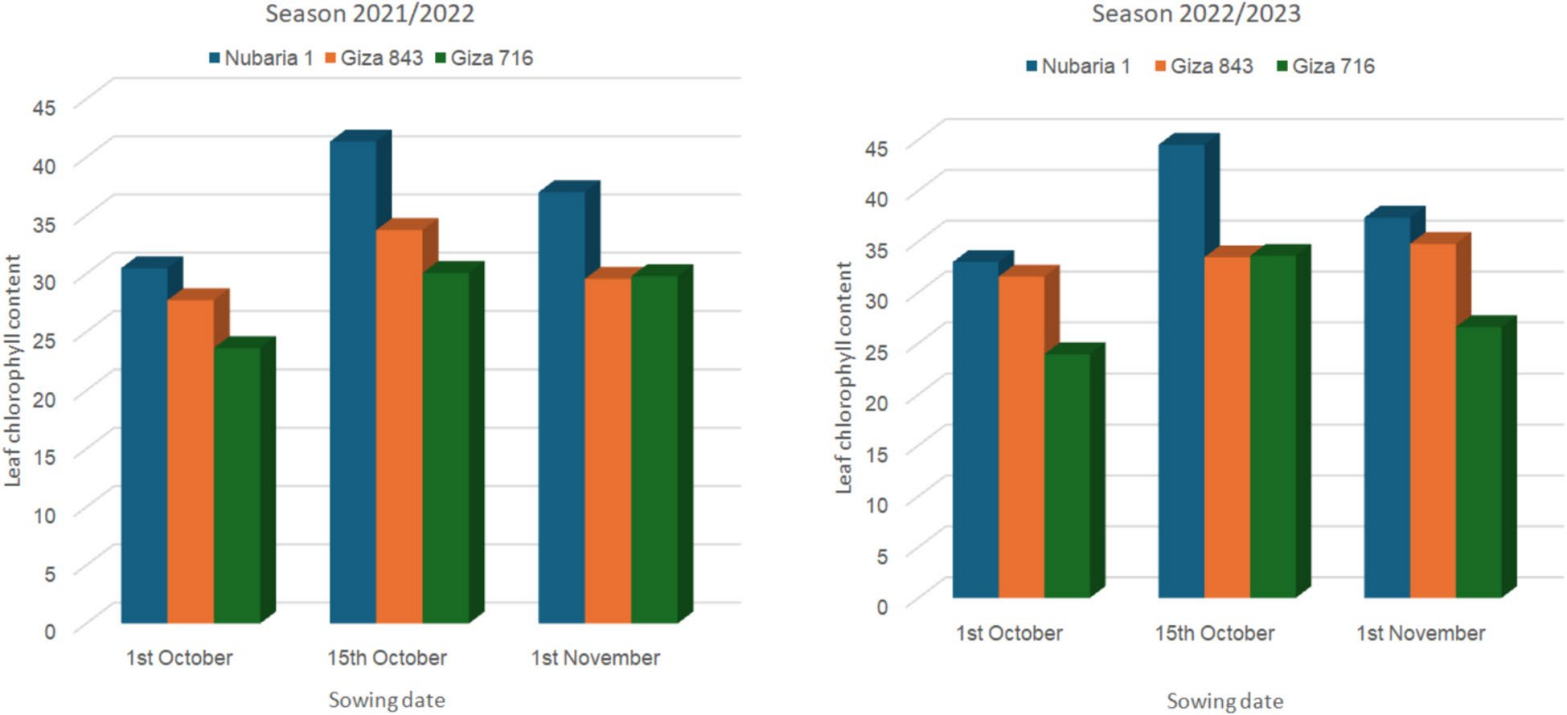
**a** Leaf *chlorophyll* content of the three faba bean varieties as affected by planting dates and their interaction in 2021/2022 season; **b** in 2022/2023 season

Figure 2a & b illustrates the effects of sowing dates on the three faba bean cultivars and their interaction with leaf chlorophyll content during the 2021/2022 and 2022/2023 seasons. Concerned about the effects of planting times on chlorophyll, the second date, mid-October, recorded the highest values of leaf *chlorophyll* readings, followed by the first of November for this trait, while the lowest values were observed on the first date, October 1 st, in each of the two seasons. Regarding the reaction of faba beans to the studied conditions, the results in Fig. 2 further demonstrate that the three cultivars varied in *chlorophyll* content, with Nubaria 1 achieving the highest leaf *chlorophyll* values, followed by Giza 843; however, the lowest values were observed in Giza 716 during both seasons.

### Shedding percentage

**Fig. 3:**
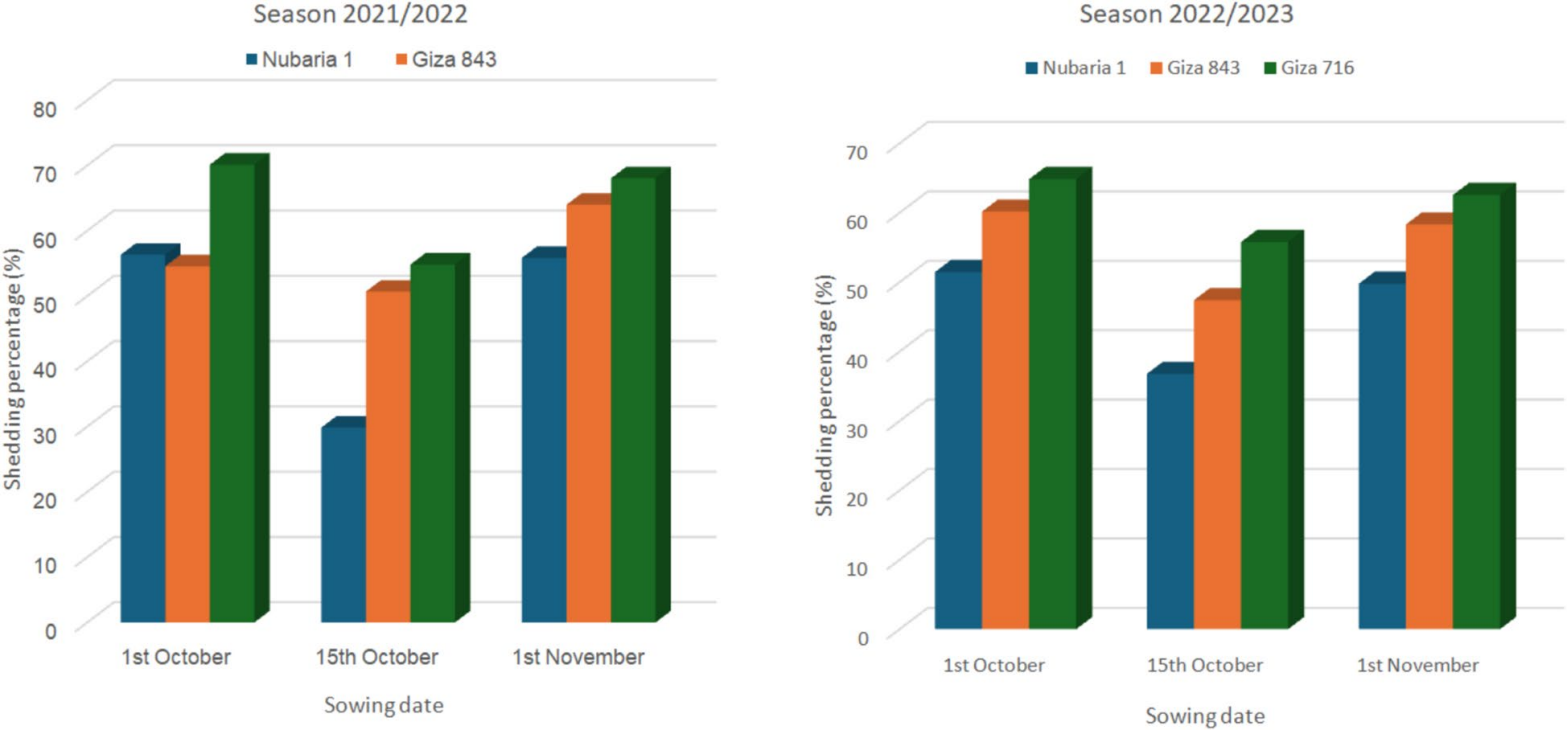
**a** Shedding percentage (%) of the three faba bean varieties as affected by planting dates and their interaction in 2021/2022 season; **b** in 2022/2023 season

**Fig. 4:**
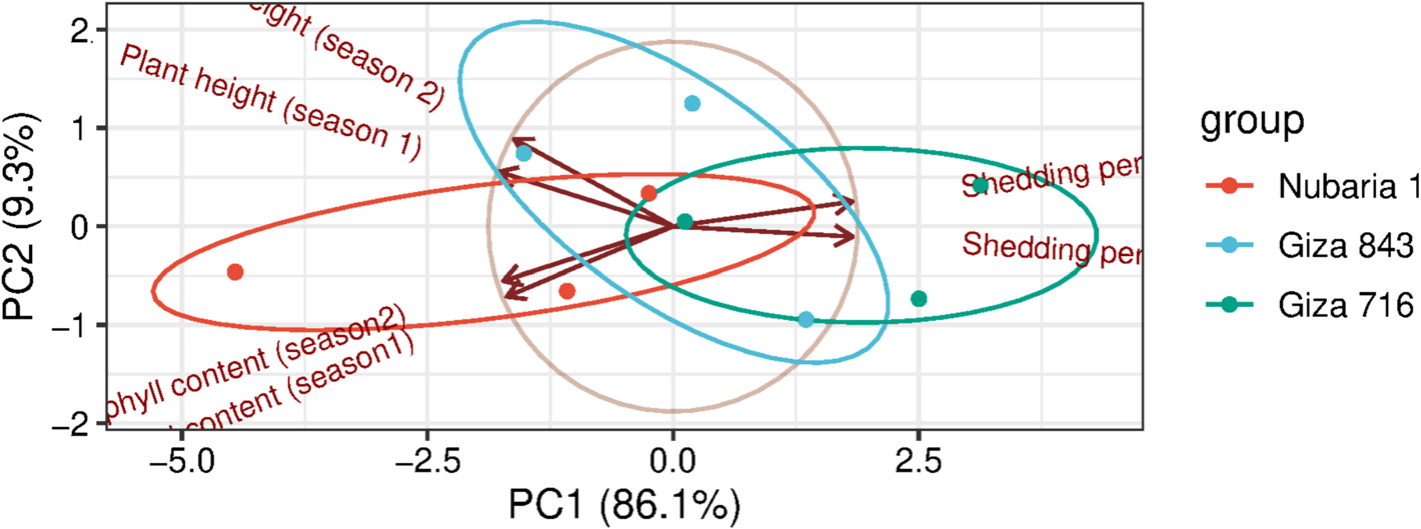
Principal component analysis of shedding percentage, plant height and chlorophyll content of the three faba bean varieties as affected by planting dates and their interaction in 2021/2022 season; **b** in 2022/2023 season

The results in Fig. 3a & b, revealed the effect of planting times on the three faba bean cultivars and their interaction on shedding percentage during the 2021/2022 and 2022/2023 seasons. The second date, 15th of October, recorded the lowest shedding percentage, followed by the 1 st of October, while November 1 recorded the lowest percentage across the two seasons. Additionally, the three cultivars differed in this trait, with Nubaria 1 showing the lowest mean values for shedding percentage, followed by Giza 843; conversely, Giza 716 exhibited the highest values in this characteristic during both seasons. Furthermore, regarding the interaction effect between sowing date and cultivars, the results indicated that planting Nubaria 1 in mid-October yielded the lowest shedding percentage, whereas sowing Giza 716 at the same time resulted in the highest percentage in the two seasons. Figure 4, principal component analysis, illustrates that three cultivars—Nubaria, Giza 716, and Giza 843—differ in terms of growth parameters, shading percentage, and chlorophyll content over two seasons.

### Identification and detection of nematode parameters

The numerous nematode species found in faba bean (*Vicia faba* L.) plants as a result of this assessment are illustrated in Tables 2 and 3; the control samples contained three distinct species of nematodes: *Xiphinema* sp., *P. brachyurus*, and *M. incognita*. There were 1520, 723, and 450 egg masses per plant, and 825, 430, and 369 galls per plant, respectively. While, in the Giza 171, just two species were recorded: *M. incognita* and *P. brachyurus*, The lowest number of galls per plant were 140 and 100, respectively, and the numbers of egg masses/plants and number of J2s/250 cm3 of soil were 80 and 60 respectively.

**Table 3:** The nematode species, gall counts, and egg masses/plant infected cultivars of faba beans (*Vicia faba* L.) season 2021/2022

Treatment	Species of nematodes	No. of gall/plant	*D %*	No. of egg masses/plant	*D %*	Number of J2s/250 g of soil	*D %*
Control	*M. incognita*	825^a^	-	1520^a^	-	320^a^	-
	*P. brachyurus*	430^b^	-	723^b^	-	289^a^	-
	*Xiphinema* sp.	369^b^	-	450^c^	-	255^b^	-
*Giza 716*	*M. incognita*	140^a^	83	80^a^	94.7	80^a^	75
	*P. brachyurus*	100^b^	76.7	60^b^	91.7	60^b^	79.2
*Giza 848*	*M. incognita*	40^b^	95	20^a^	98.6	80^a^	75
	*Xiphinema* sp.	100^a^	72.8	20^a^	95.5	80^a^	68.6
	*P. brachyurus*	0	100	20^a^	97.2	60^b^	79.2
*Nubaria 1*	*P. brachyurus*	40^b^	90.6	0	100	60^a^	79.2
	*Xiphinema* sp.	0	100	0	100	40^b^	84.3

Data are means of 10 replicates; means with the same letter(s) in each column are not significantly different at *P* ≤ 0.05. D%: decrease

**Fig. 5:**
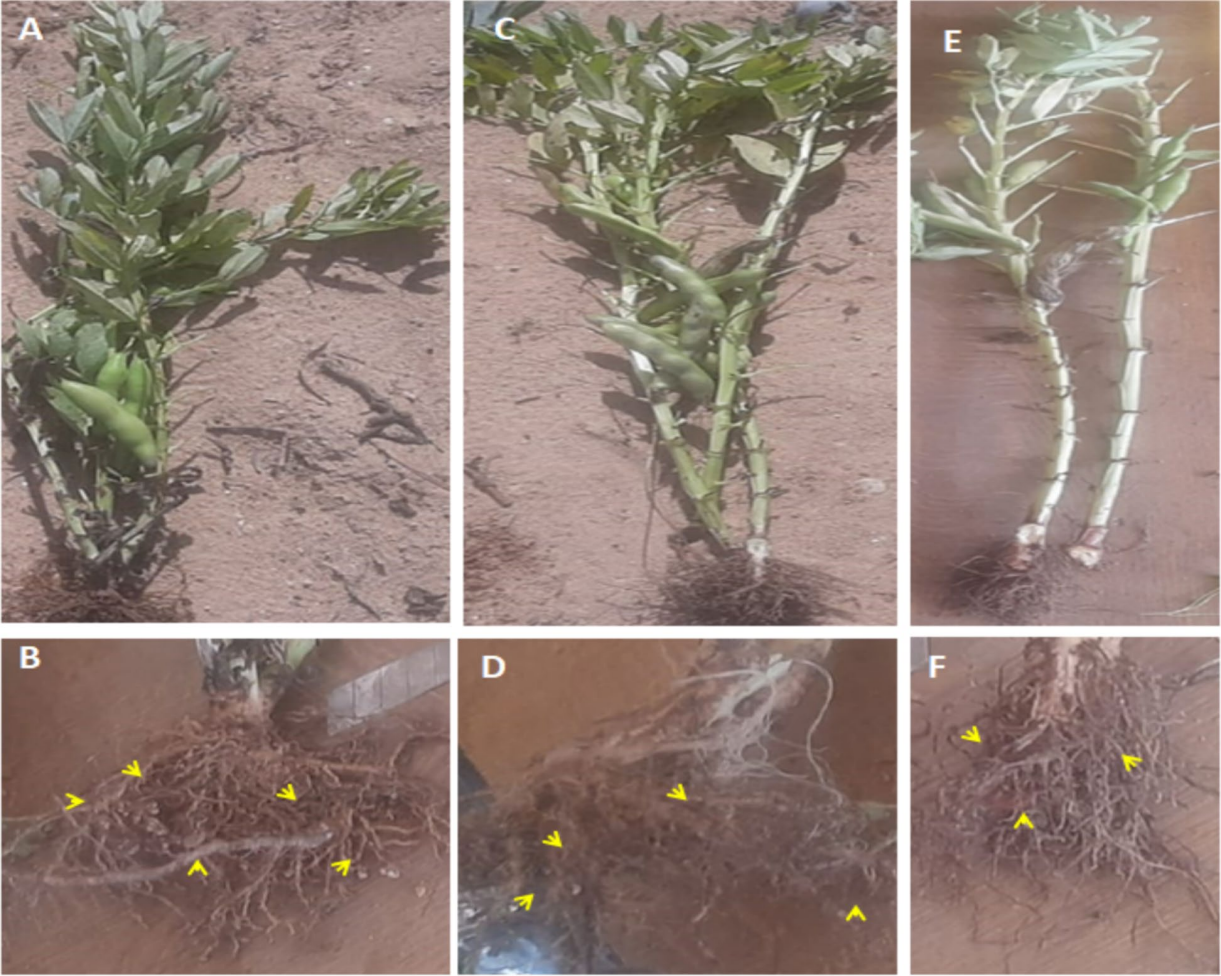
Nematode symptoms on faba bean cultivars, **A **and **B**: Giza 716; **C** and **D**: Giza 848; **E** and **F**: Nubaria1

Notably, *M. incognita*, *P. brachyurus*, and *Xiphinema* sp. were the three species found in Giza 848 with 20 egg masses per plant, in addition to 40, 100, and 0 galls per plant, respectively. 68.6, 79.2, and 79.2% decrease% at the number of J2s/250 cm3 of soil, respectively. Finally, Nubaria 1 displayed two species with 40 and 0 gall per plant, respectively: *P. brachyurus* and *Xiphinema* sp. Figure 5.

### The induced/suppressed genes in the faba bean plants with different agricultural times

Gene expression at the mRNA level can be compared and differences between any two samples can be found using the differential display (DD-PCR) technique. Using cDNA produced from faba bean tissue that had its mRNA extracted, a PCR differential display analysis was carried out using four distinct primers.

**Fig. 6:**
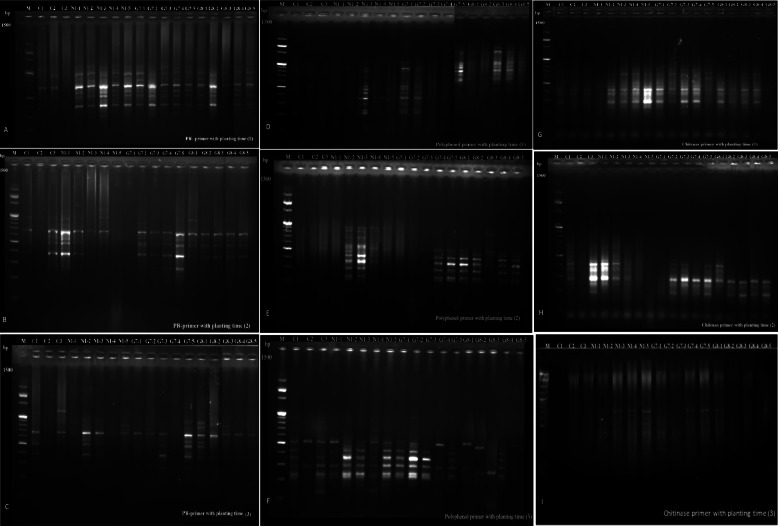
Agarose gel electrophoresis (1.5%) TBE buffer stained with ethidium bromide, showing differential display PCR using **A**, **B** and **C** PR1 primer with planting time 1, 2, and 3. **D**, **E** and **F** ppo primer with planting time 1, 2, and 3. **G**, **H** and **I** Chitinase primer with planting time 1, 2, and 3

A thorough analysis of the amplified cDNA revealed variations in gene occurrence and density in bands found in the plant under examination and up-down regulated bands in the examined samples and not in the control. Figure 6(A1) displays the data with polyphenol primer (PPO) in 117 bands during the initial sowing period on October 1, 2021. Thirty of the 117 bands are monomorphic, and 87 are polymorphic. Additionally, there was 74.35% genetic polymorphism. Particularly for Nubaria 1 in the third month following sowing, Giza 716 in the fifth month, and Giza 843 in the third, the treated samples displayed distinct fragments when compared to the control samples. When the primer polyphenol was sown a second time on October 15, 2021, Fig. 6(A2) revealed 97 bands in total. Thirteen of the 97 bands are monomorphic, and eighty-four are polymorphic, with 86.59% genetic polymorphism. Additionally, the analyzed samples displayed distinct fragments and elevated gene expression in comparison to control samples, particularly for Nubaria 1 in the second and third months following planting. The primer polyphenol data from the third sowing cycle on November 1, 2021, revealed 121 bands, of which 28 are monomorphic and 93 are polymorphic at 76.85% genetic polymorphism. Notable All the control and other samples examined of faba bean produced fragments after the high molecular weight of 700 pb. Particularly for Nubaria 1 in the second and fifth months following sowing, the studied samples displayed distinct fragments and elevated gene expression compared with the control samples Fig. 6(A3).

Regarding the second primer, PR-1 (F), which was initially sown on October 1, 2021, a total of 157 bands were found, 70 of which were monomorphic and 87 of which were polymorphic, with the genetic polymorphism at 55.41%. All the control and treated faba bean samples produced similarity in the range of 45%, Fig. 6(B1).

The data attained for the second time of sowing on October 15, 2021, revealed 83 bands: 31 bands monomorphic and 52 bands polymorphic at 62.65% genetic polymorphism. This data shows the control Giza 716 cultivars don’t produce any fragments compared with the other two controls, Nubaria 1 and Giza 843. The results showed that Nubria 1 in the fifth month and Giza 716 in the first month didn’t detect any fragments Fig. 6A, B, and C. The data attained for the second time of sowing on October 15, 2021, revealed 83 bands: 31 bands monomorphic and 52 bands polymorphic at 62.65% genetic polymorphism. This data shows the control Giza 716 cultivars don’t produce any fragments compared with the other two controls, Nubaria 1 and Giza 843. The results showed that Nubria 1 in the fifth month and Giza 716 in the first month didn’t detect any fragments Fig. 6(B 3). When the third primer, Chitenas primer, was first sown on October 1, 2021, Fig. 6D, E and F revealed that of the 86 bands, 48 were monomorphic and 38 were polymorphic and showed different fragments compared with the control group, which revealed the lowest number of bands these data, Nubaria 1 in the fifth month (before harvest), displayed the highest number of DNA fragments comparing with the other faba bean samples and control.

Finally, Fig. 6G, H and I shows that the second time of sowing on October 15, 2021, produced 72 polymorphic bands with a genetic polymorphism of 77.77%. Based on these data, the results showed that the control of Nubaria 1 and Giza 843 cultivars in the first month detected a high number of fragments and an increase in gene expression tested by Chitenas primer, while the control Giza 716 and Nubria 1 before harvest and Giza 716 in the first month did not record any response to the same primer. Finally, the other faba bean samples detected many fragments.

### Gene expression quantification (Q-PCR)

**Fig. 7:**
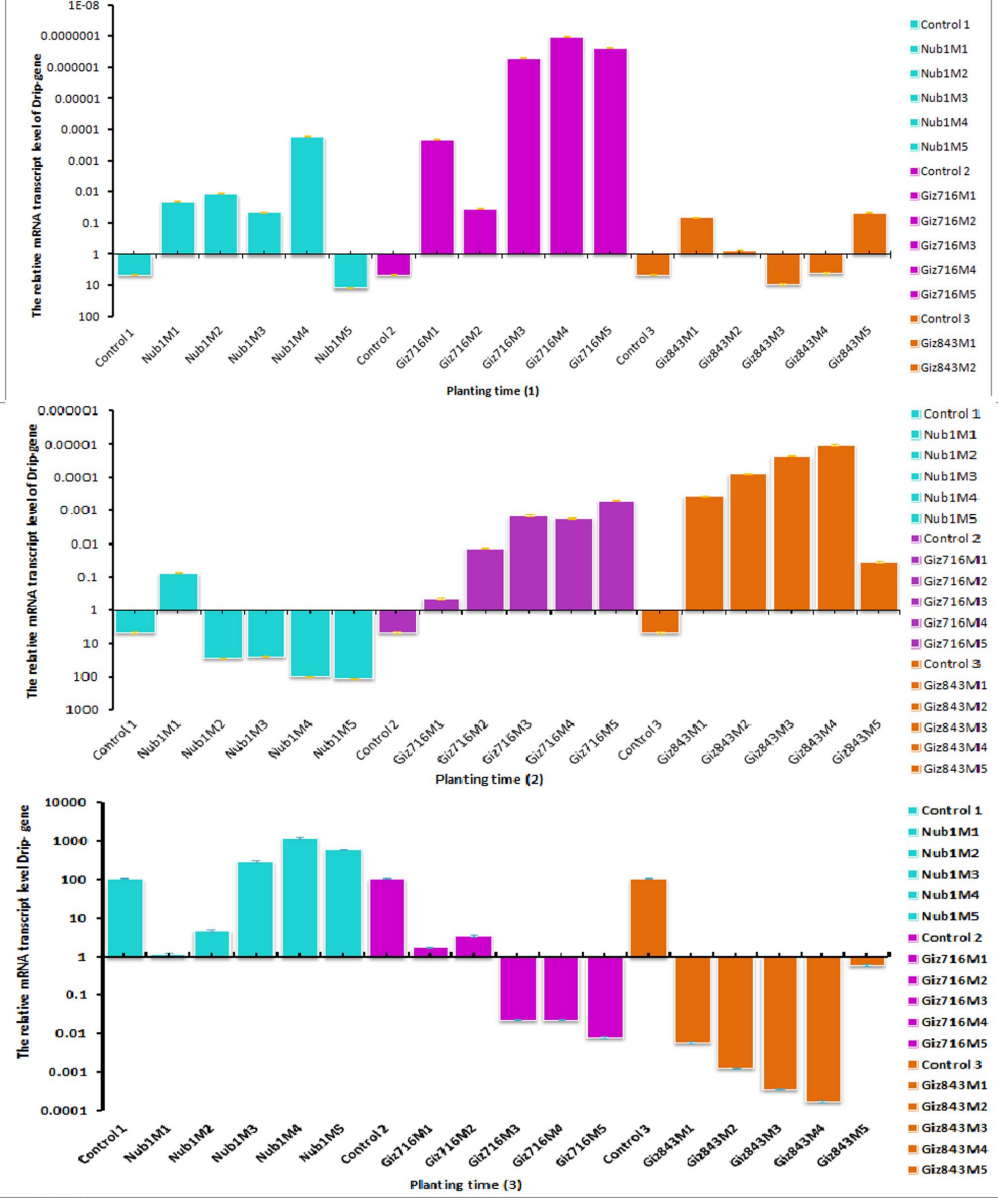
The different in gene expression of faba bean cultivars tested by Drip gene during the first planting time (1): 1 October 2021; planting time (2): 15 October 2021; planting time (3):1 November 2021

The Drip gene test revealed that the three-control group's up-regulated (turned on) genes were Nubria 1, Giza 716, and Giza 843, as well as Nubria 1 (fifth month) and Giza 843 (third and fourth month), which were the first instances of high activity and increased gene expression under open field conditions (1 October 2021). In contrast, the other faba bean treatments exhibited down-regulation compared with the control (Fig. 7, A). For the same gene, the Drip gene under the second time (15 October 2021) control group showed high activity and an increase in gene expression as up-regulated genes (turned on) with Nubria 1 (from second to five months). However, during this sowing period, the other treatments showed no response to this gene. The results detected the lowest gene expression was observed for the Giza 843 during the different growth stages, as found in Fig. 7B. When it comes to the Drip gene in Fig. 3C for the third time (1 November 2021), the control group exhibited high activity and an increase in gene expression as up-regulated genes (turned on) with Nubria 1 (during the growth stage) and Giza 716 (first and second month), whereas the other treatments showed no response with this gene during this sowing period. The results detected the lowest gene expression observed for the Giza 843 during the different growth stages.

**Fig. 8:**
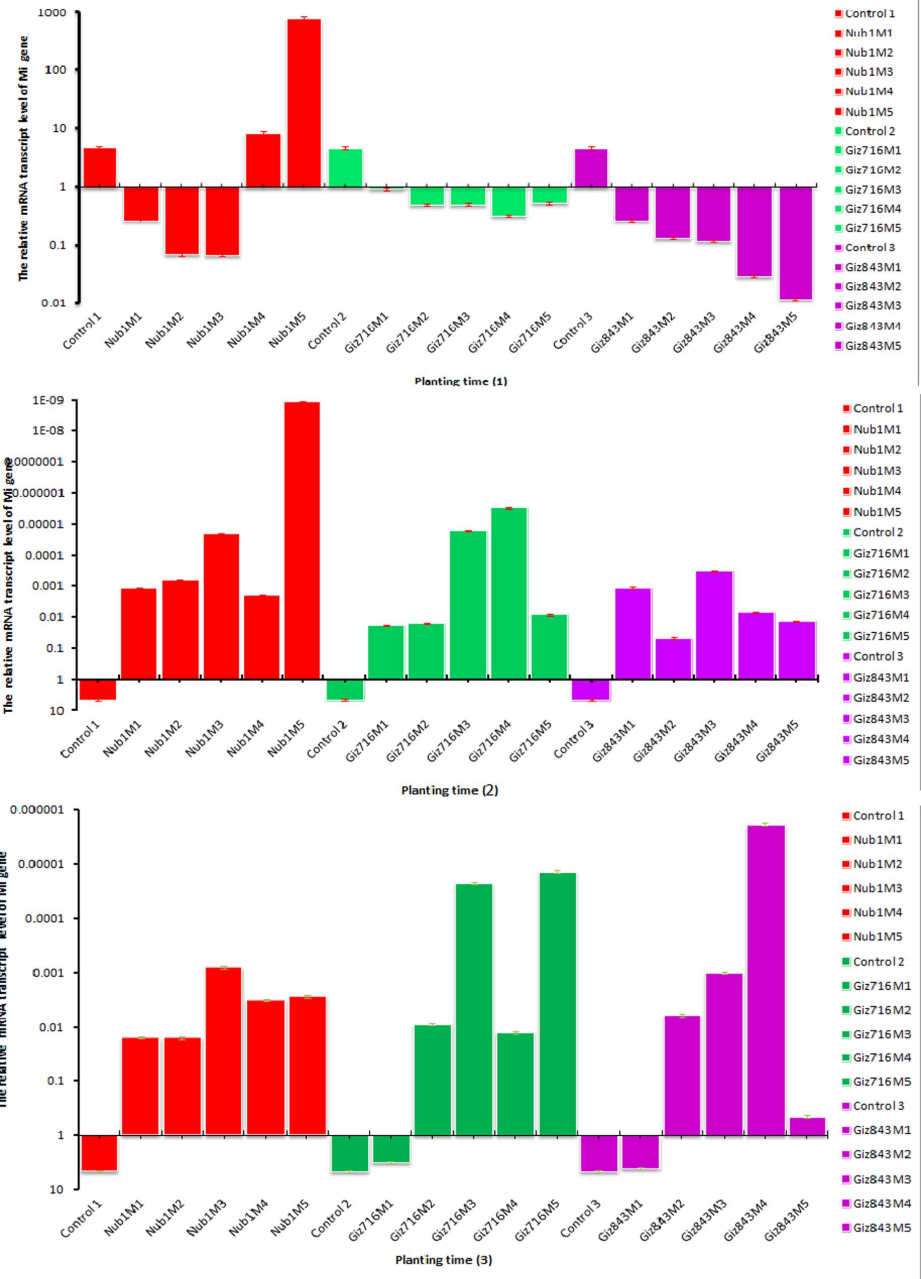
The different in gene expression of faba bean cultivars tested by Mi-CYP gene during the first planting time (1): 1 October; (2): 15 October; (3): 1 November

In the case of gene MI, Nubaria 1 (five months after sowing) showed significantly different gene expression from the other treatments. Upon subsequent planting, Nubaria showed moderate gene expression compared to the control group four months later. In contrast, the data in Fig. 8(1) indicated that Giza 843 was down-regulated. Unfortunately, aside from the control group, which displayed the lowest gene expression, none of the treatments in Fig. 8(2) for the gene M1 exhibited any expression at all on the second sowing date. Gene expression for Giza 716 and Giza 843 (first month after sowing) was observed in addition to the third sowing time. Nevertheless, Giza 716 (3 and 5 months) and Giza 843 (4 months after sowing) have a high down-regulated (turned off) gene, as found in Fig. 8(3).

## Discussion

This study aimed to investigate the impact of sowing date on nematode infestation and ascertain the degree of plant-parasitic nematode infestation in them important faba bean-producing region at the ideal planting time.

The findings showed that different genotypes of faba bean cultivars reacted differently to various environmental conditions as well as planting time, indicating the significance of evaluating genotypes in various environments to determine the optimal cultivar composition for a given environment. Similar findings were made by [57] who found that the first-order interaction—that is, sowing date x cultivars—had a significant impact on all investigated vegetative and yield characteristics. Therefore, the combined effect of the two factors under study will affect the value recorded for characters as parasitic nematode invasion. Figure 1, shows how the sowing date affected the nematode parameter studied traits. The environmental factors (temperature, humidity, and day length) varied during crop growth and at the time of sowing in various natural photothermal environments. It is possible to interpret the observed variation in the cultivars'studied characteristics between early and optimum sowing dates as a result of both weather and sowing date variations. The Giza 716 cultivar was infected by the nematodes *M. incognita* and *P. brachyurus*, while the Giza 848 cultivar was infected by *Xiphinema* sp., *M. incognita*, and *P. brachyurus*. These nematodes showed high values in the early sowing date. On the other hand, the Nubaria 1 cultivar simultaneously displayed low values for nematode infection. Likewise, [58] found that sowing early through 31 st October produced considerably higher biomass and yield except for"Azad P-1"and"Pb-89,"the"*Palam priya*"variety produced the same yield on October 31 of the first year in both years. [59] noticed variances in the outcomes of various varieties on various dates of sowing in the yield of seed and composition traitsc [60] found that, sowing on 10th November have the highest seed yield. [61], found that, late sowing dates reduced the amount of diseases infection, while the highest seed yield was obtained from optimum sowing date. These results agree with [46] who obtained that an early sowing date resulted in higher infection rates than the ideal sowing date [6, 62–64], all reported similar significant first-order interactions, i.e., sowing date x genotypes. Root galls are the primary sign of root-knot nematode infection by intricate physiological and biochemical alterations brought on by parasites. According to [65], these alterations cause cell hypertrophy and hyperplasia, which affect the root's ability to absorb water and nutrients and consequently hinder plant growth and yield.

The high relative temperatures that prevailed during the various stages of the common bean growing season (March–April)–with average temperatures of 23 and 25°C compared to 16 and 20°C during the fall—may have contributed to nematodes'impact on the growth and physiological characteristics of common beans grown in the early spring. The low temperature (less than 20°C) also reduced nematode activity and their ability to attack plants and reproduce [66].

Additionally, bean plants mature before the first nematode generation emerges due to the delayed nematode life cycle, which can take longer than two months [67]. This significantly harms the hosts [68]. High temperatures significantly influence several crop plants'expression of resistance to root-knot nematodes.

When temperatures rise above a certain threshold for heat stability—which is established by the effects of temperature on the nematode and/or the crop plant—plant resistance to plant-parasitic nematodes generally decreases. According to [69], the common bean showed less resistance to *M. incognita* at 28°C than at 16 or 21°C.

The second aim of this study is to investigate gene expression and plant defence. The study found that when faba bean plants were planted on October 10th, the expression of defence-related genes PPO, chitinase, and Pr3 increased. Chitinase is essential for tearing down chitin, a crucial structure for wall cells [70].

The study found that when faba bean plants were planted on October 10th, the expression of defence-related genes PPO, chitinase, and POD increased. Chitinase is essential for tearing down chitin, a crucial structure for wall cells.

When plant tissues are subjected to many different stresses, over expression of the antioxidant stress marker gene POD helps reduce reactive oxygen species (ROS). The increased activity of POD and PPO enzymes supported this defence response. When faba bean plants were planted in October, the severity and incidence of disease were lessened thanks to the up-regulation of these genes. Furthermore, the build-up of nematotoxic phenolic compounds in plants may prevent *M. incognita* from growing and spreading between cells.81.

Generally, a plant's stress causes the synthesis of protective secondary metabolites that aid in pathogen defence. These mechanisms include cell wall lignifications, production of nematicidal substances, stimulation of phytoalexins and PR-proteins, and increased expression of defence-related genes [71]. In the mid-October planting, the plant immune system appears to be activated against soil-borne parasites like RKNs through the up-regulation of ppo, pod, and Pr3-responsive genes.

Additionally, it was shown that down-regulation of genes encoding for antioxidant enzymes and increased glucanase and chitinase activities were involved. Although systemic immunity is provided, its impact most likely diminishes as the nematode infection worsens. An extra tactic in the integrated pest management strategy. However, tactics that rely on the activation of plant innate immunity appear to have the potential to be effective in agriculture against plant parasitic nematode damaging invasion.

## Conclusions

The findings of this study indicate that Nubaria 1 cultivar, when planted on October 15th, exhibits reduced susceptibility to nematode infestation, providing a potential non-chemical solution for faba bean production.

## Supplementary Information

Supplementary Material 1.

## Data Availability

The corresponding author can provide all of the datasets used in the analysis of the current study upon reasonable request.
