# Supplementary figures and images for: The impact of planting times and cultivars on the excitation of innate-immunity response against populations and severity of plant parasitic nematodes in faba bean (Vicia faba L.) field
Source: BMC Plant Biol. 2025 Sep 16;25:1204. doi: 10.1186/s12870-025-07148-8 (PMC12439394; doi:10.1186/s12870-025-07148-8)

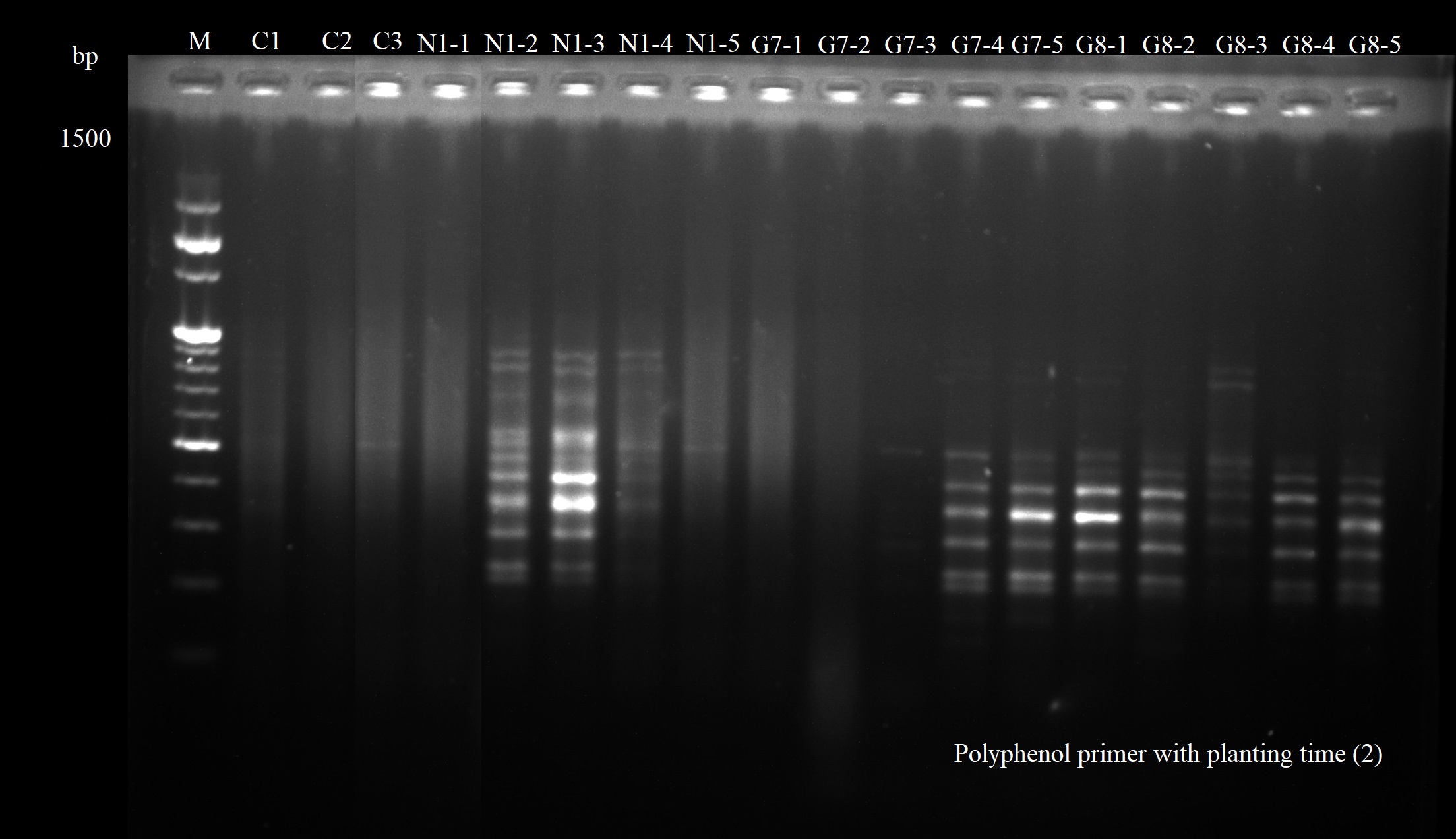

Supplement: Supplementary file 1 — Supplementary Material 1. [file 12870_2025_7148_MOESM1_ESM.zip › 14~30 poly phenol.jpg]

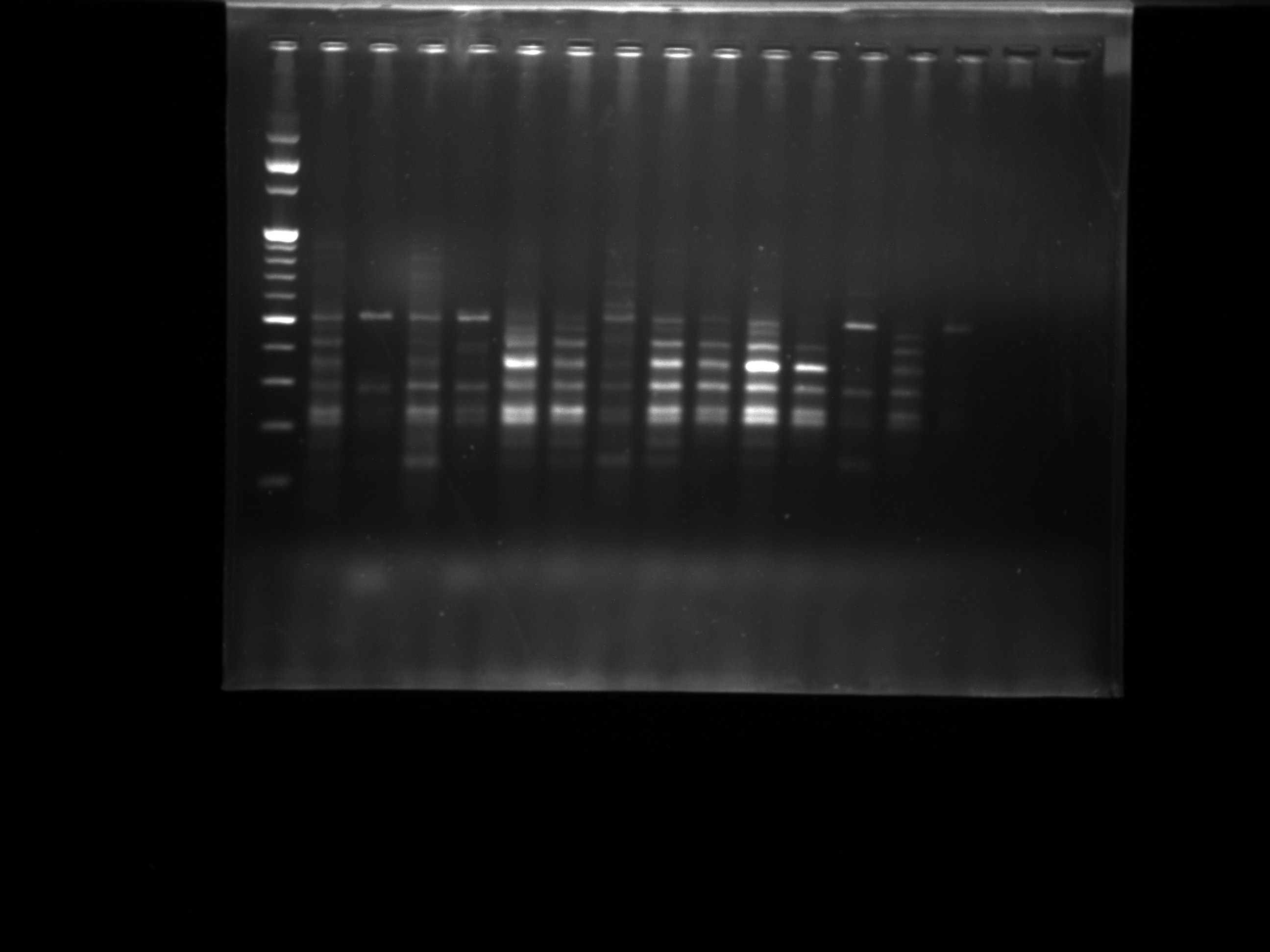

Supplement: Supplementary file 1 — Supplementary Material 1. [file 12870_2025_7148_MOESM1_ESM.zip › 31~45 polyphenol.jpg]

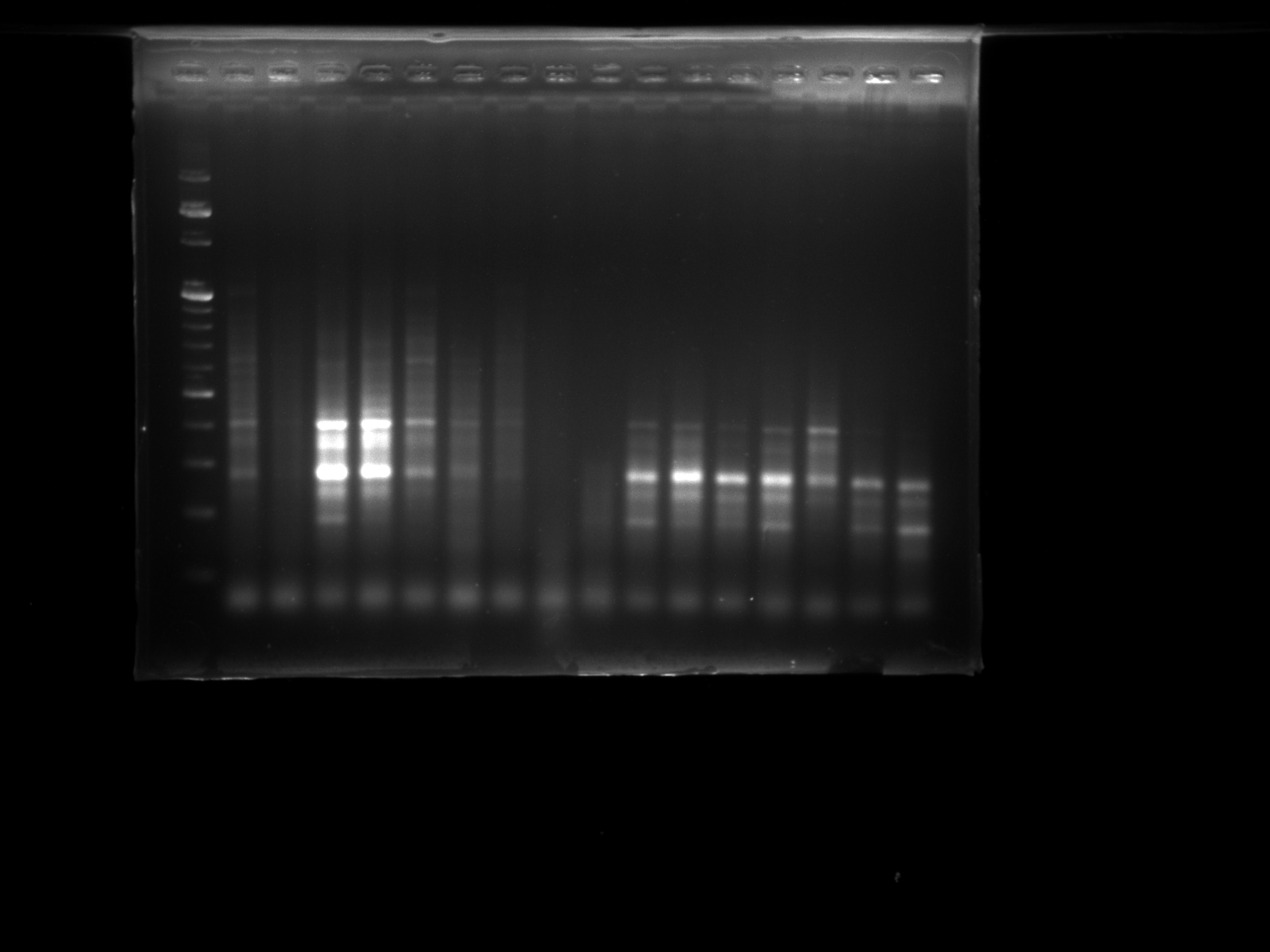

Supplement: Supplementary file 1 — Supplementary Material 1. [file 12870_2025_7148_MOESM1_ESM.zip › chiti 3R (14~29) 1.jpg]

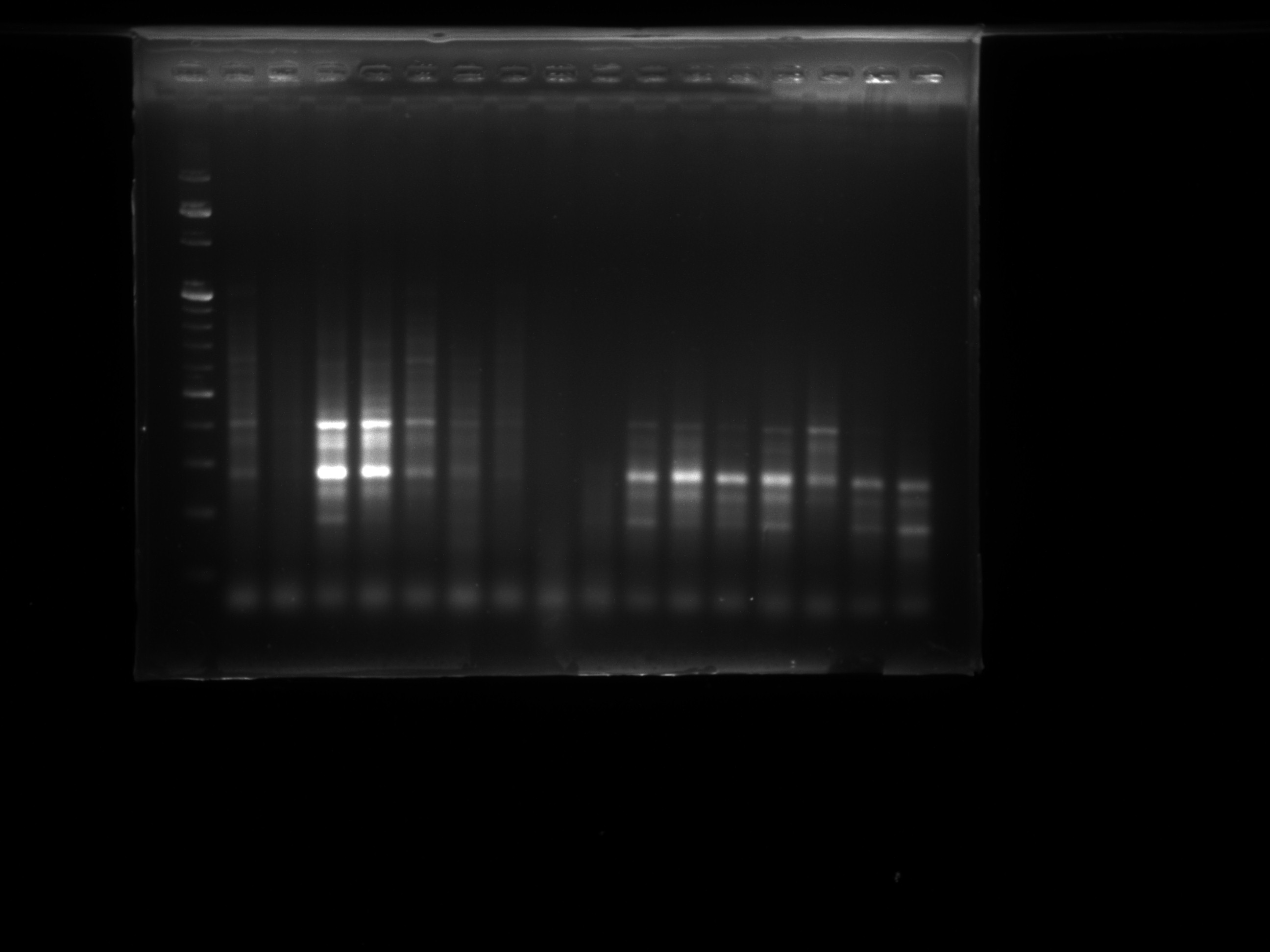

Supplement: Supplementary file 1 — Supplementary Material 1. [file 12870_2025_7148_MOESM1_ESM.zip › chiti 3R (14~29).jpg]

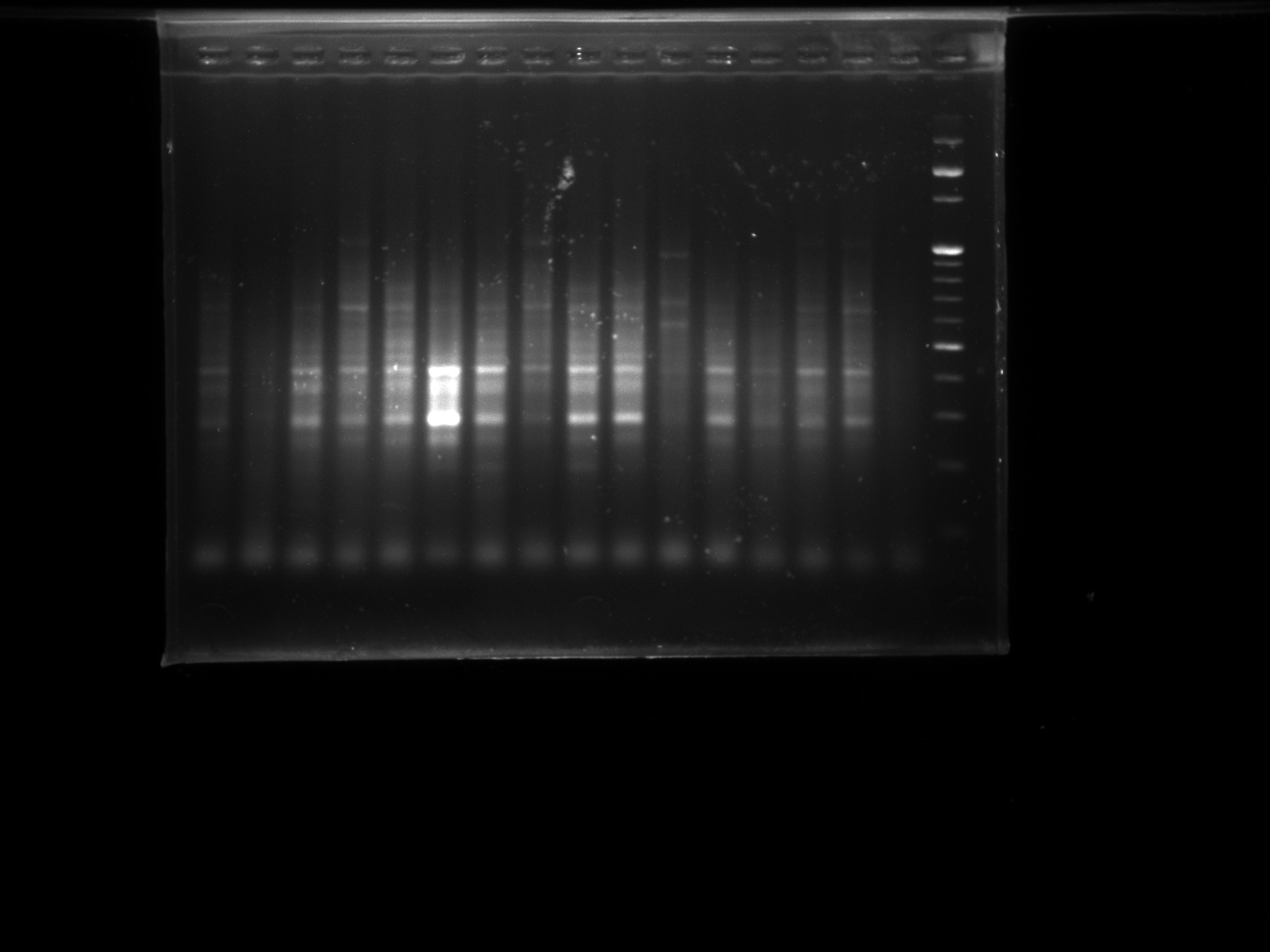

Supplement: Supplementary file 1 — Supplementary Material 1. [file 12870_2025_7148_MOESM1_ESM.zip › chiti 3R (v1~13) 1.jpg]

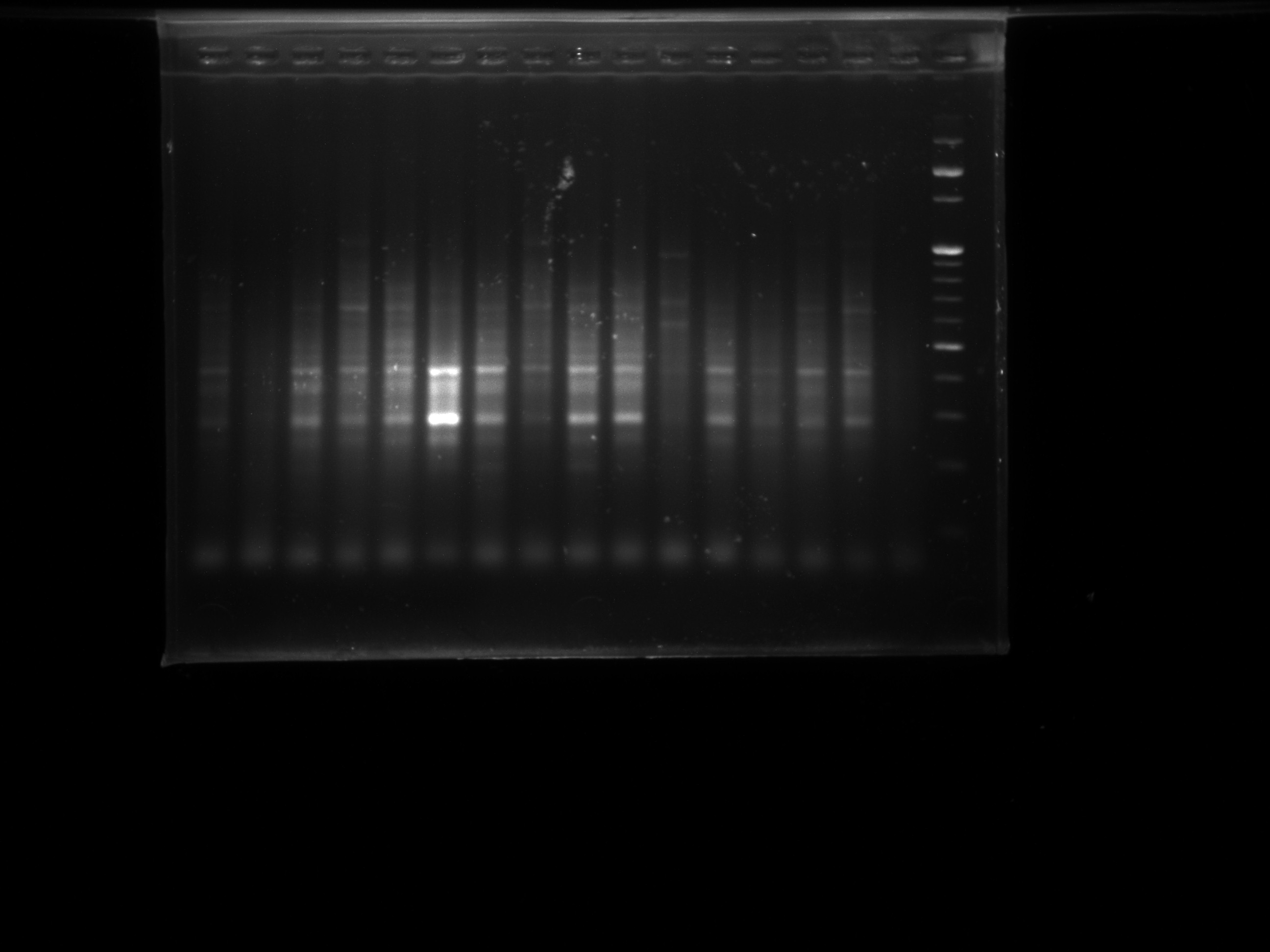

Supplement: Supplementary file 1 — Supplementary Material 1. [file 12870_2025_7148_MOESM1_ESM.zip › chiti 3R (v1~13).jpg]

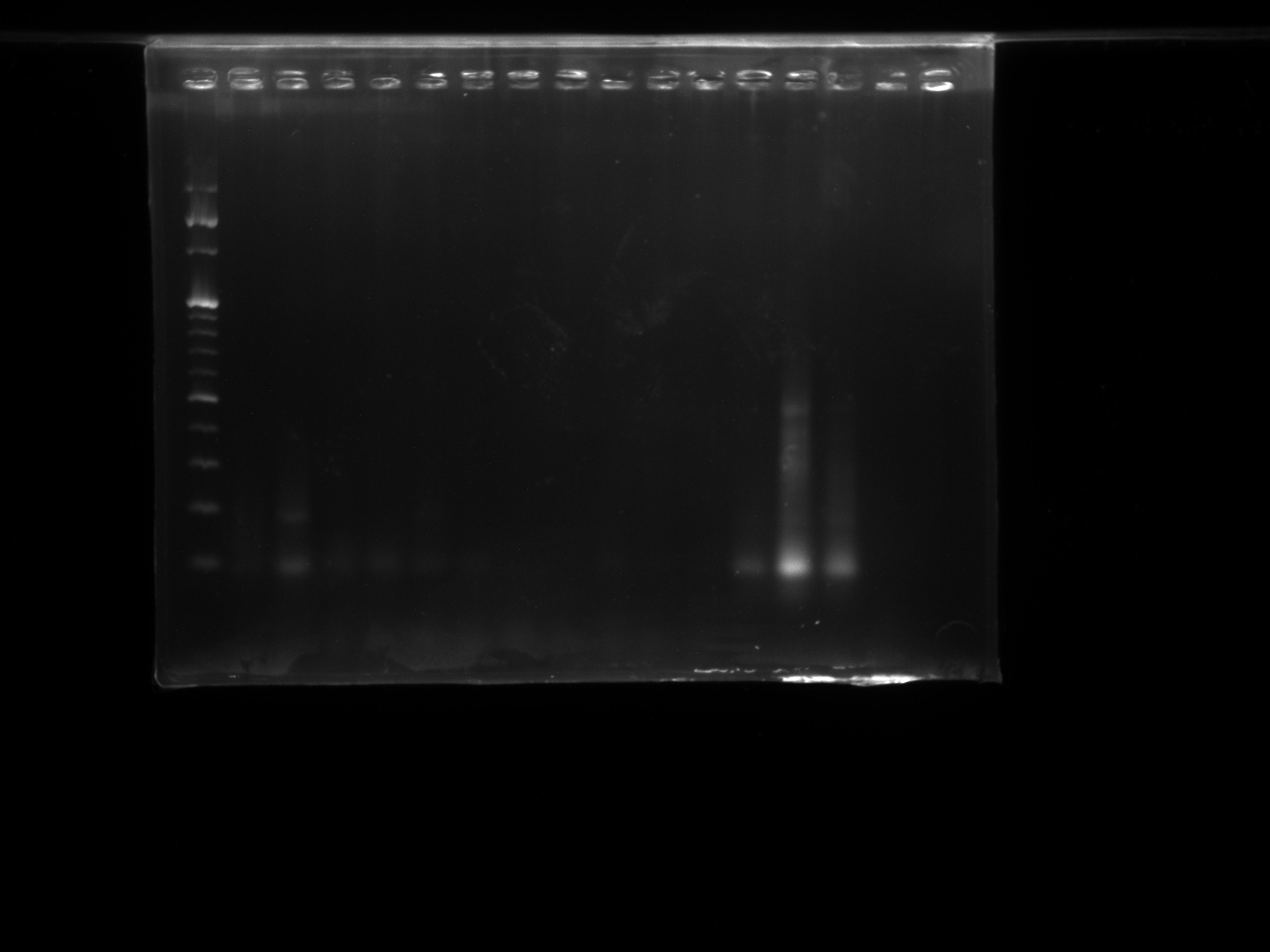

Supplement: Supplementary file 1 — Supplementary Material 1. [file 12870_2025_7148_MOESM1_ESM.zip › missing 2.jpg]

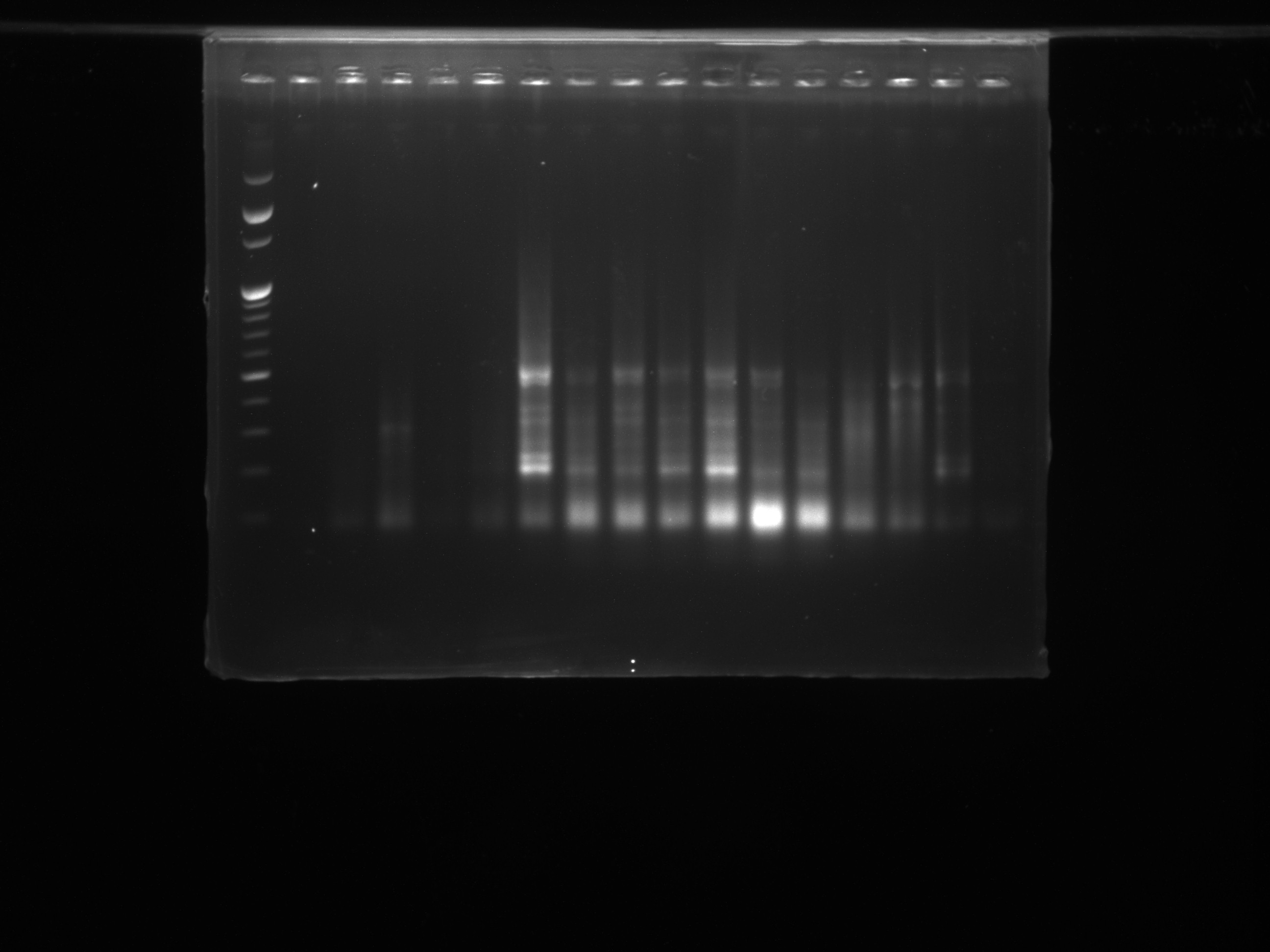

Supplement: Supplementary file 1 — Supplementary Material 1. [file 12870_2025_7148_MOESM1_ESM.zip › missing blots.jpg]

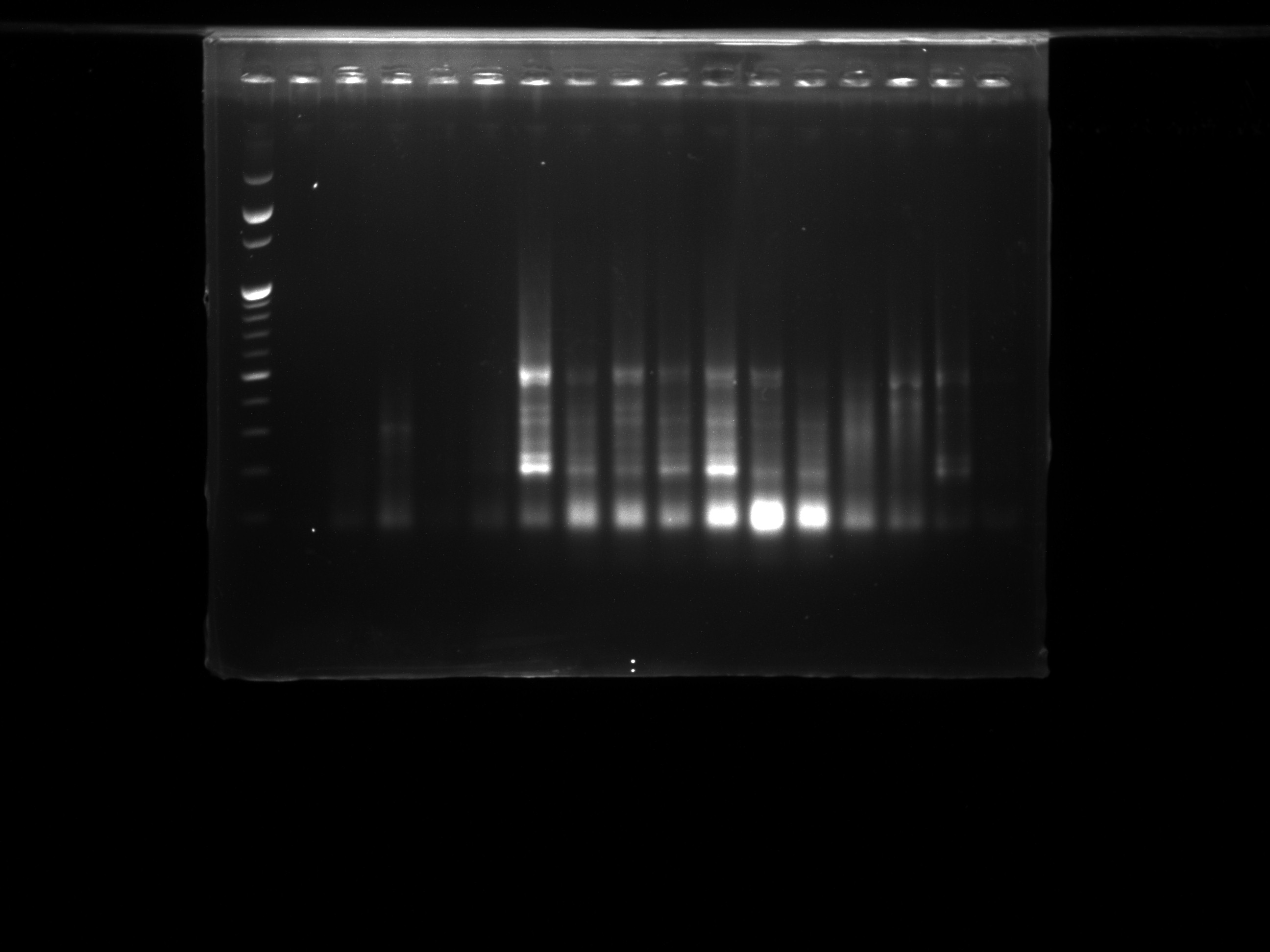

Supplement: Supplementary file 1 — Supplementary Material 1. [file 12870_2025_7148_MOESM1_ESM.zip › missing lines.jpg]

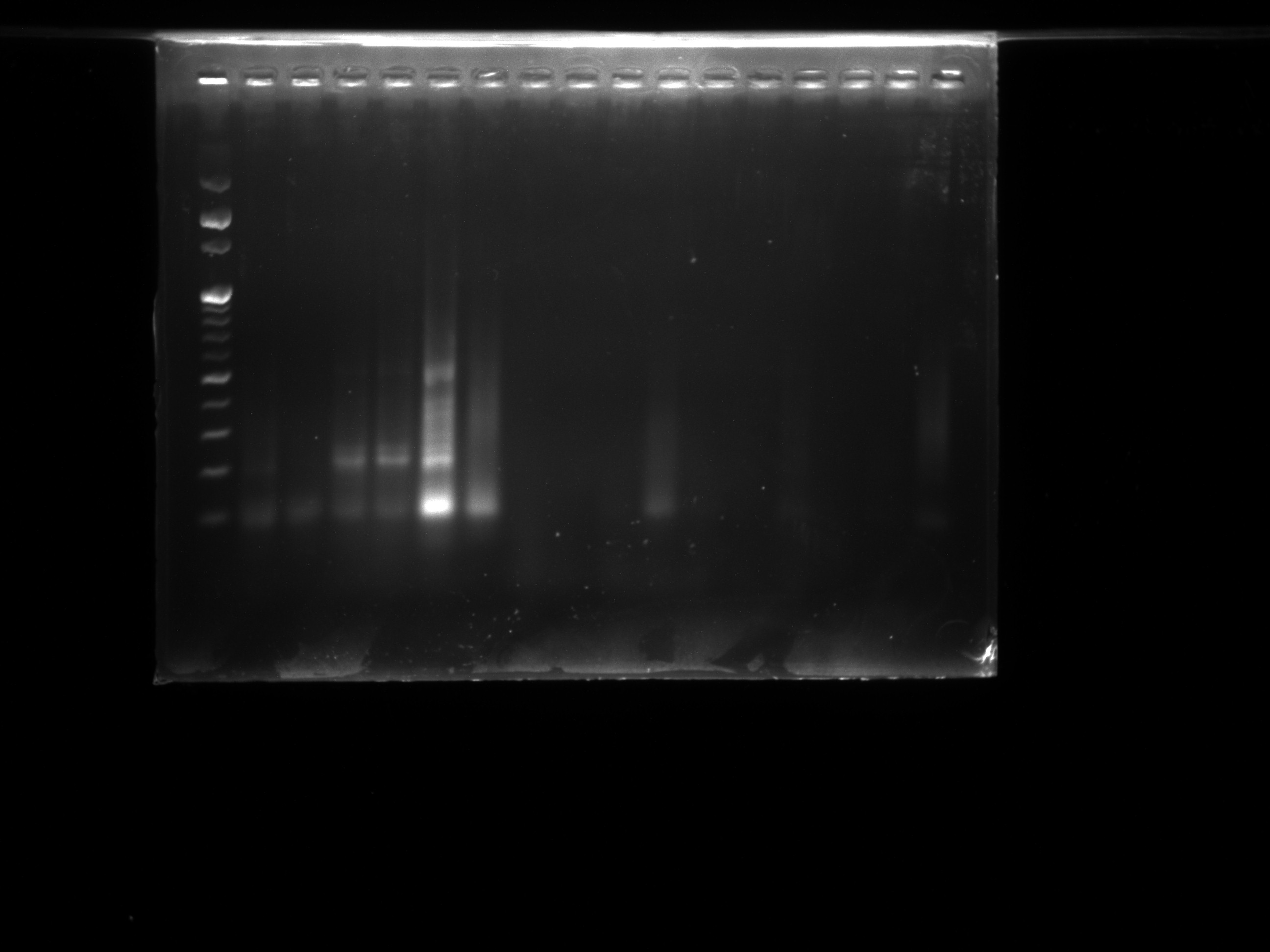

Supplement: Supplementary file 1 — Supplementary Material 1. [file 12870_2025_7148_MOESM1_ESM.zip › missing.jpg]

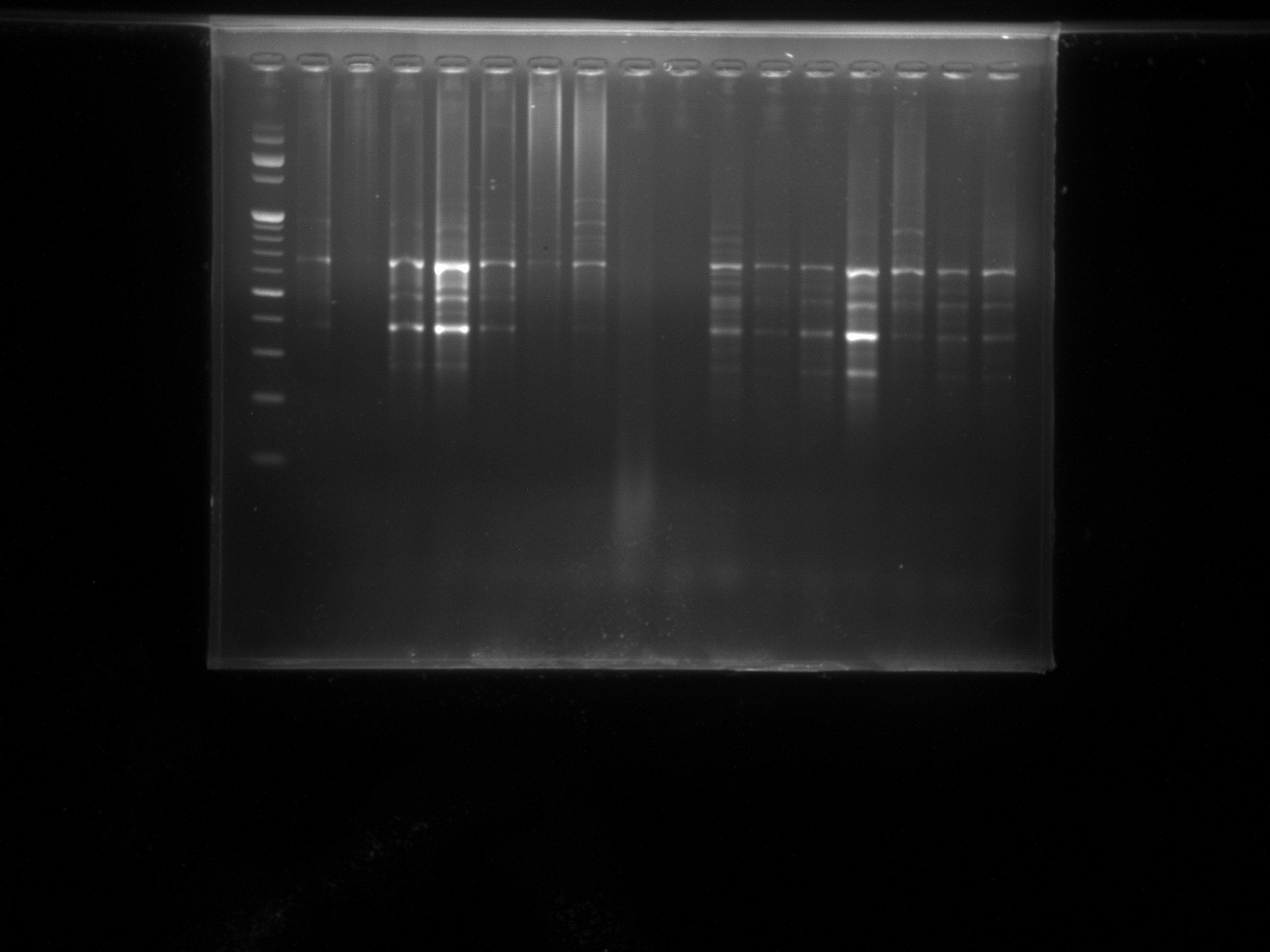

Supplement: Supplementary file 1 — Supplementary Material 1. [file 12870_2025_7148_MOESM1_ESM.zip › PR 14~29 1.jpg]

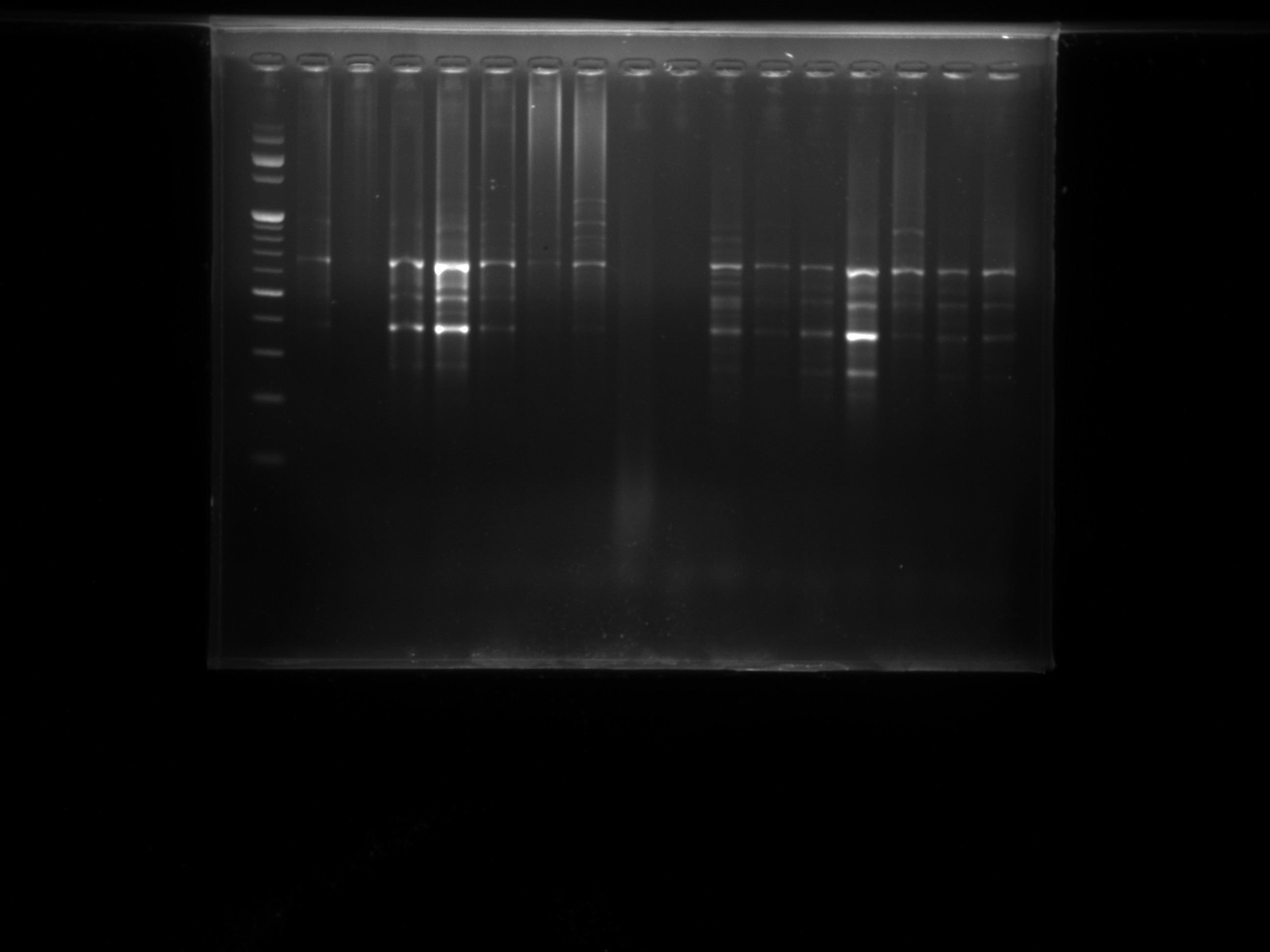

Supplement: Supplementary file 1 — Supplementary Material 1. [file 12870_2025_7148_MOESM1_ESM.zip › PR 14~29 2.jpg]

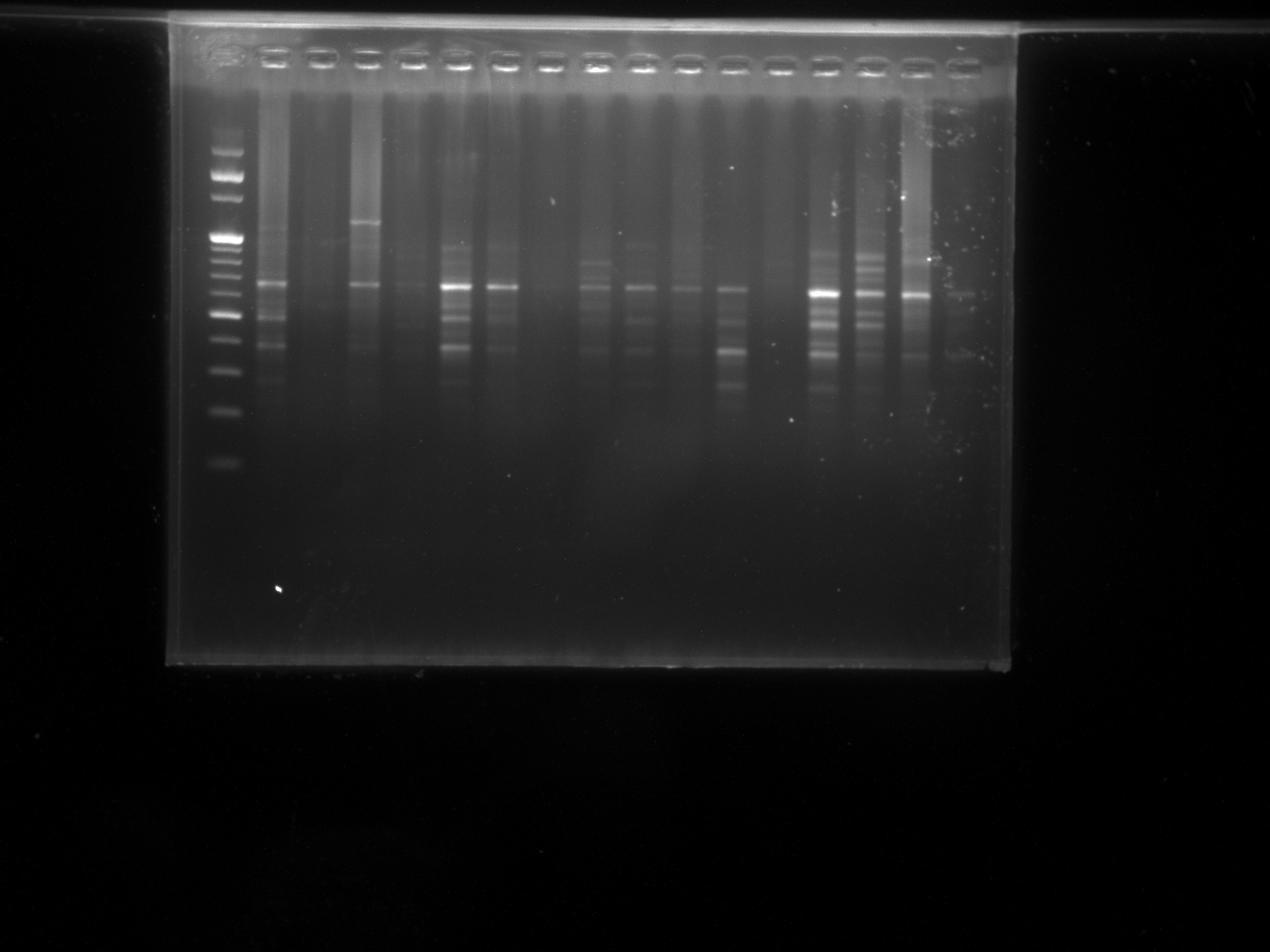

Supplement: Supplementary file 1 — Supplementary Material 1. [file 12870_2025_7148_MOESM1_ESM.zip › PR 30~45 1.jpg]

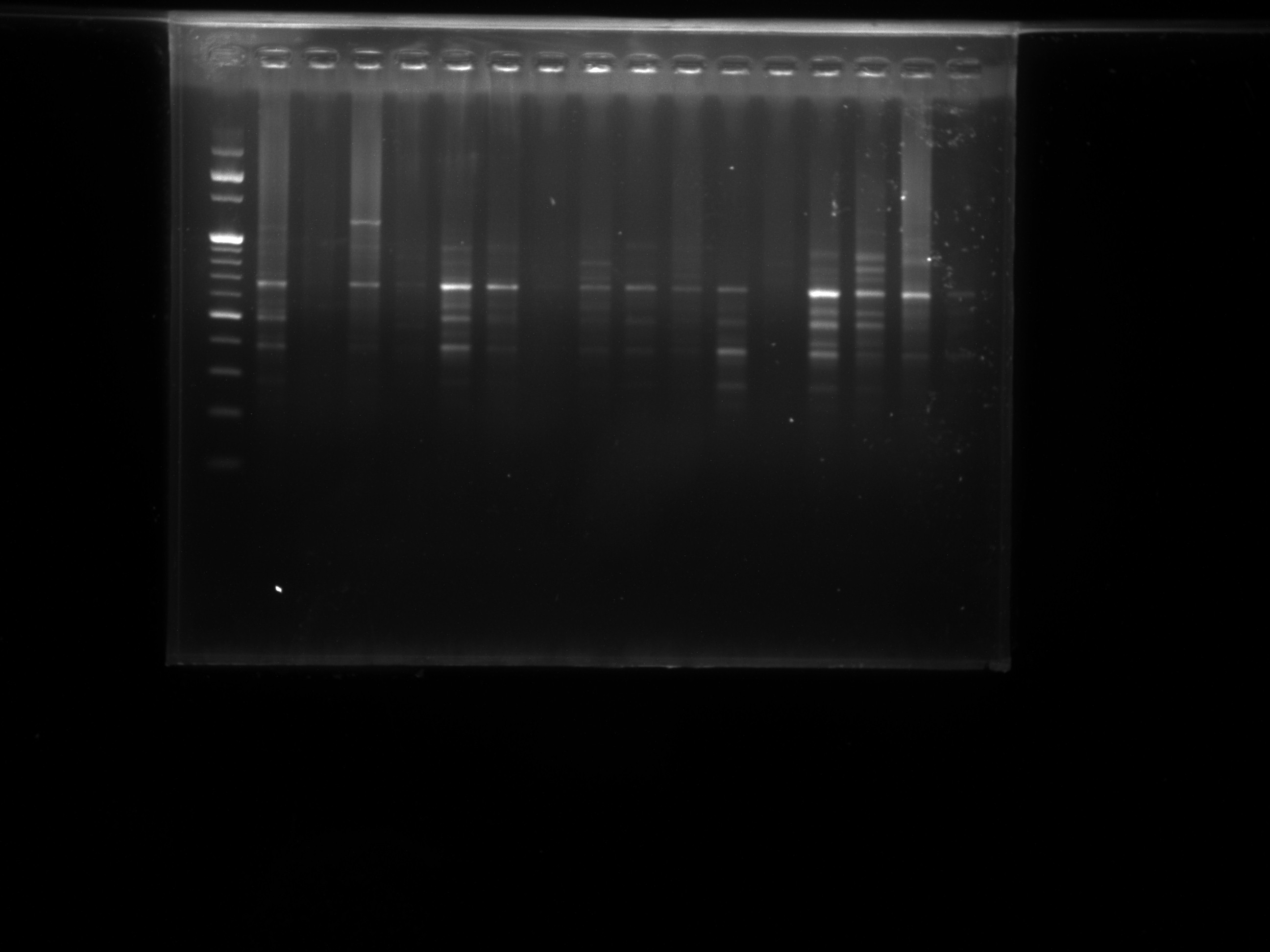

Supplement: Supplementary file 1 — Supplementary Material 1. [file 12870_2025_7148_MOESM1_ESM.zip › PR 30~45 2.jpg]

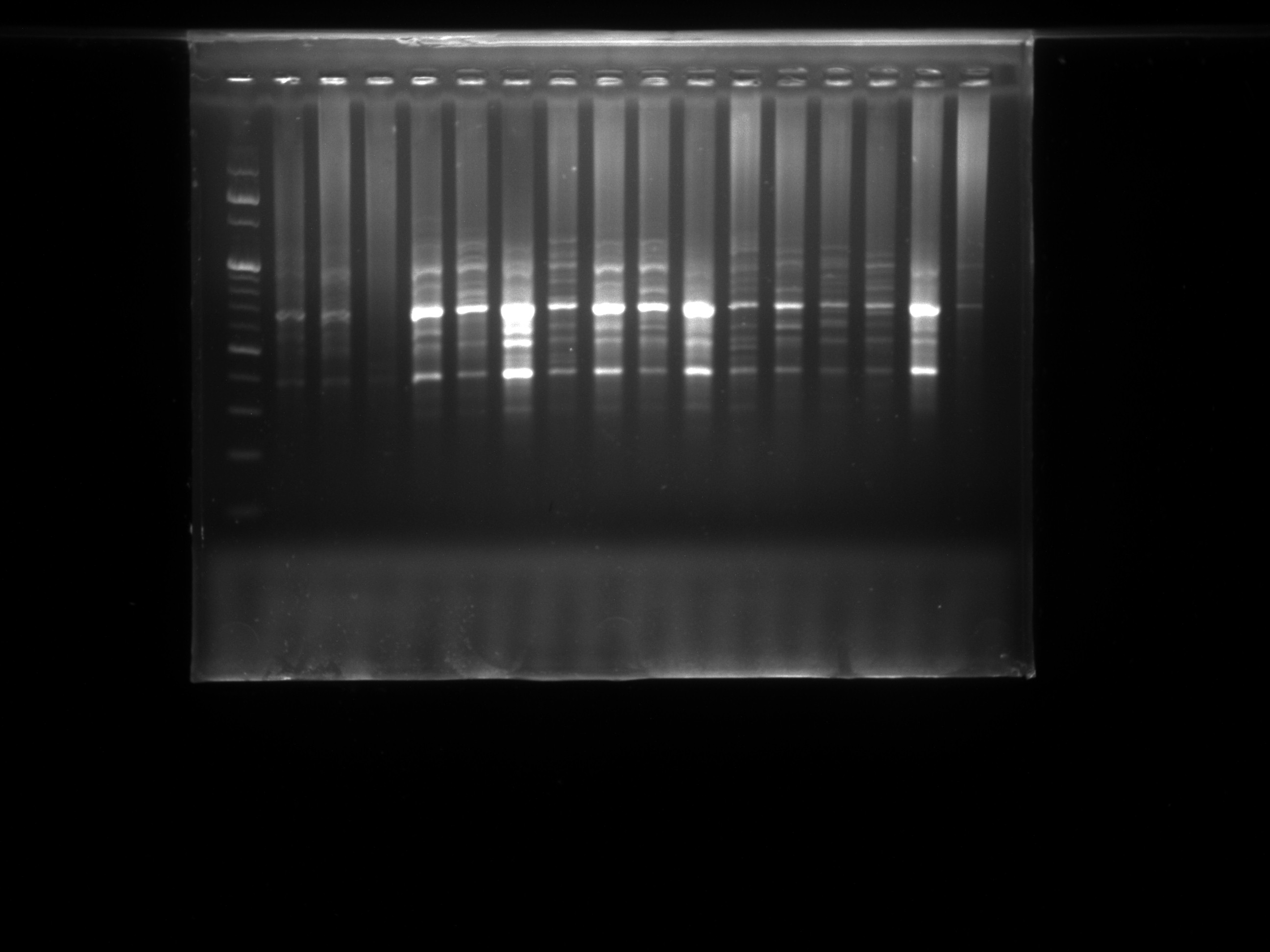

Supplement: Supplementary file 1 — Supplementary Material 1. [file 12870_2025_7148_MOESM1_ESM.zip › PR v1~13 2.jpg]

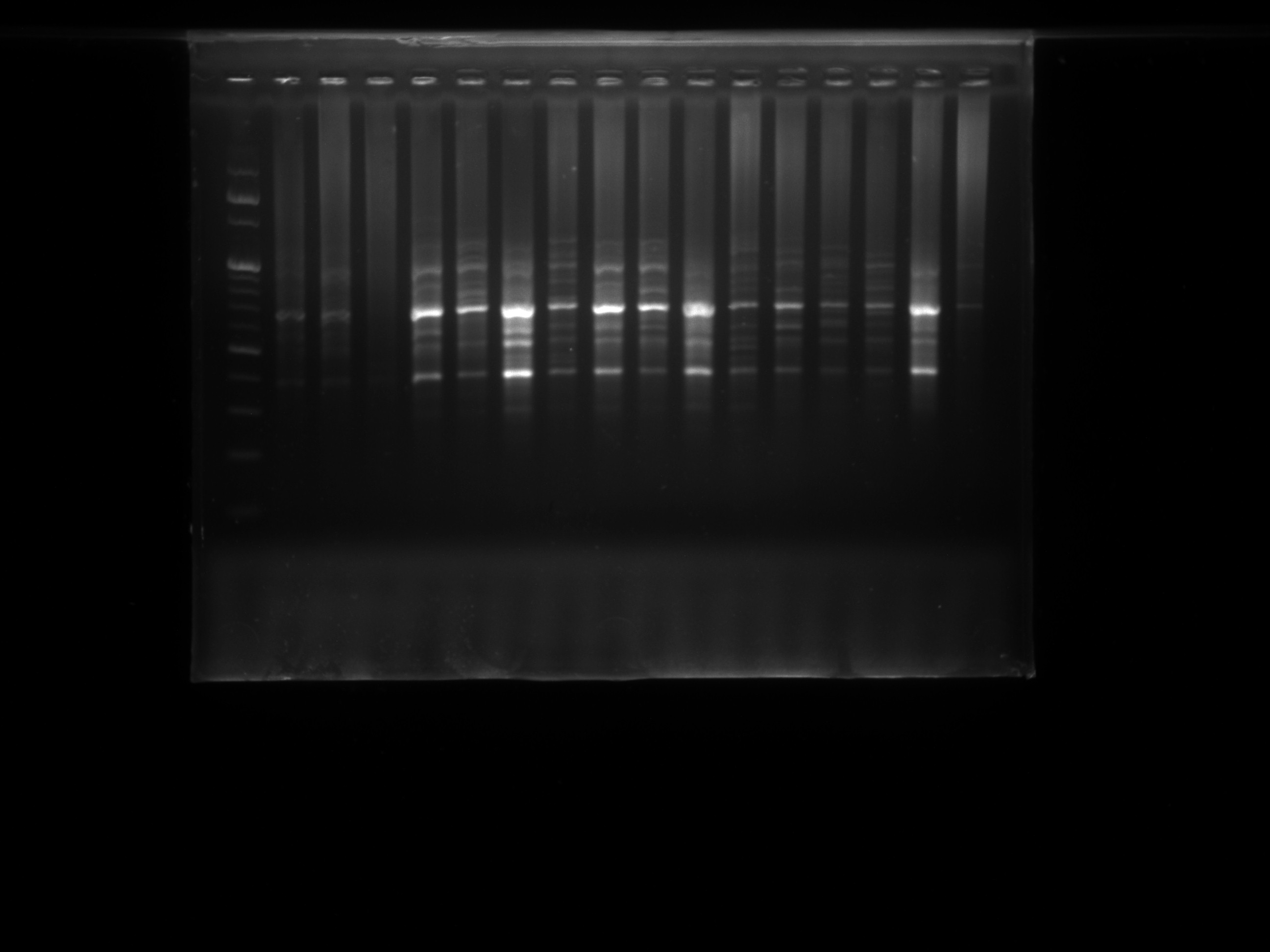

Supplement: Supplementary file 1 — Supplementary Material 1. [file 12870_2025_7148_MOESM1_ESM.zip › PR v1~13 1.jpg]

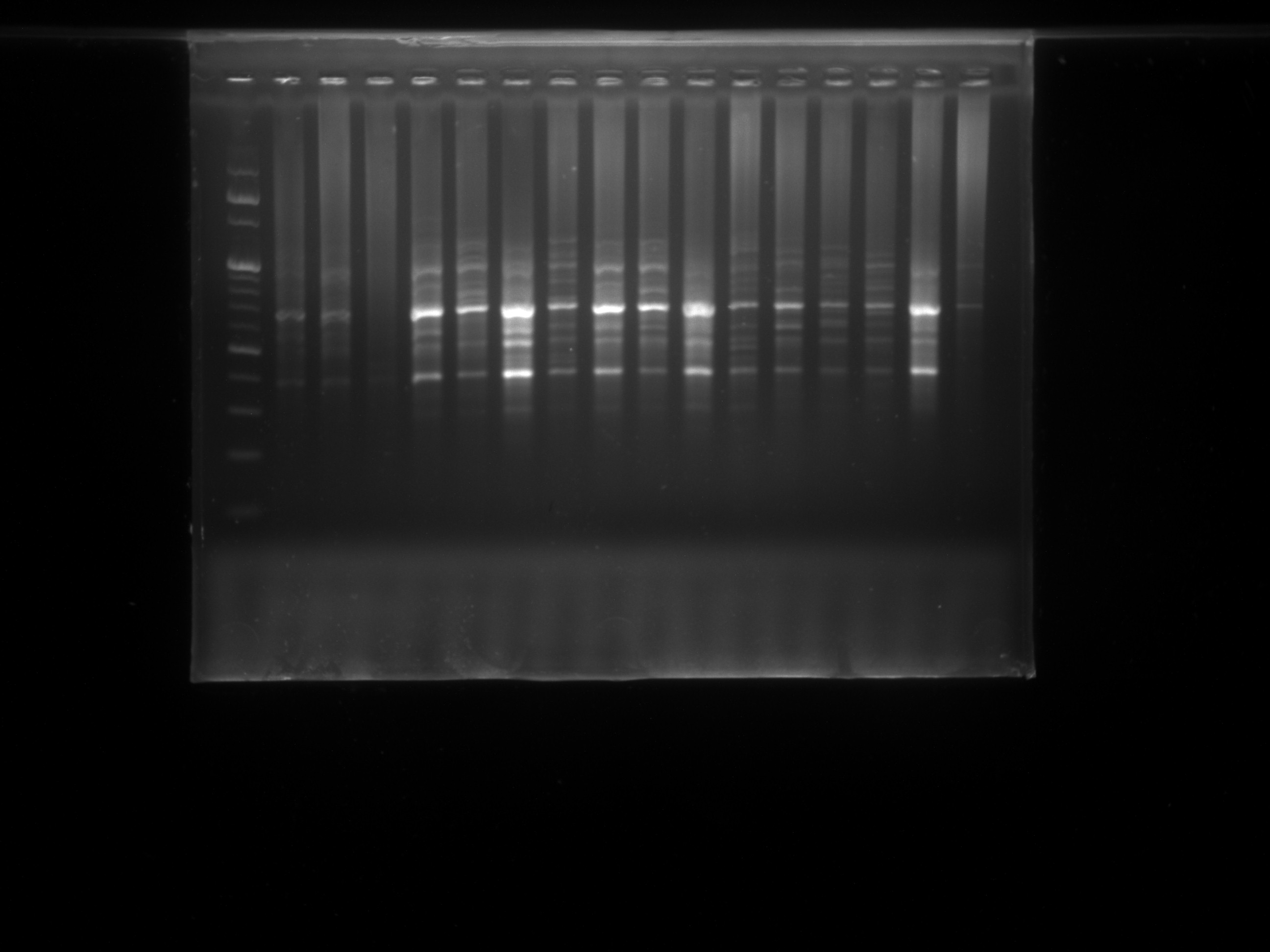

Supplement: Supplementary file 1 — Supplementary Material 1. [file 12870_2025_7148_MOESM1_ESM.zip › PR v1~13.jpg]

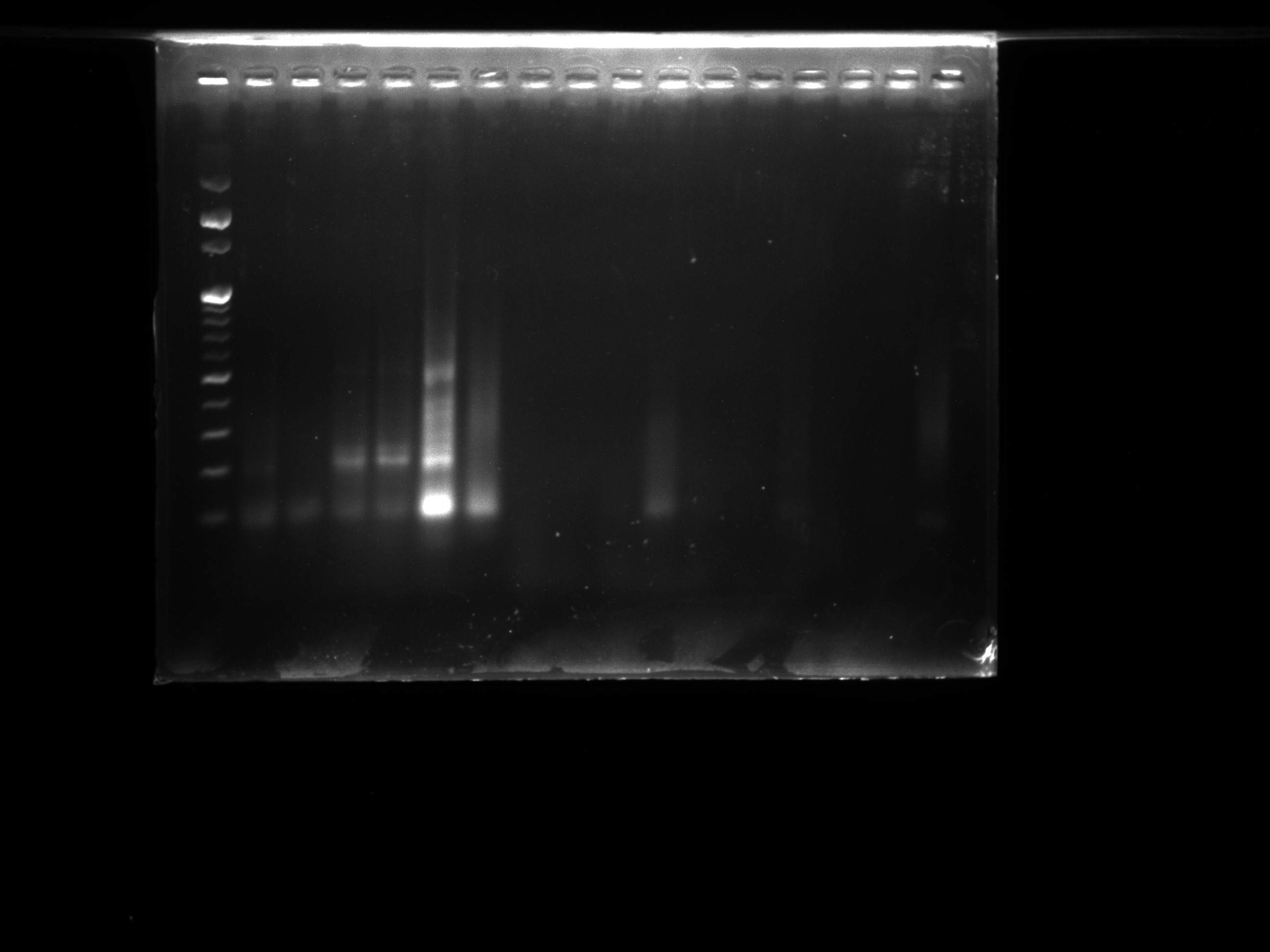

Supplement: Supplementary file 1 — Supplementary Material 1. [file 12870_2025_7148_MOESM1_ESM.zip › THR 3F (14~29) 2.jpg]

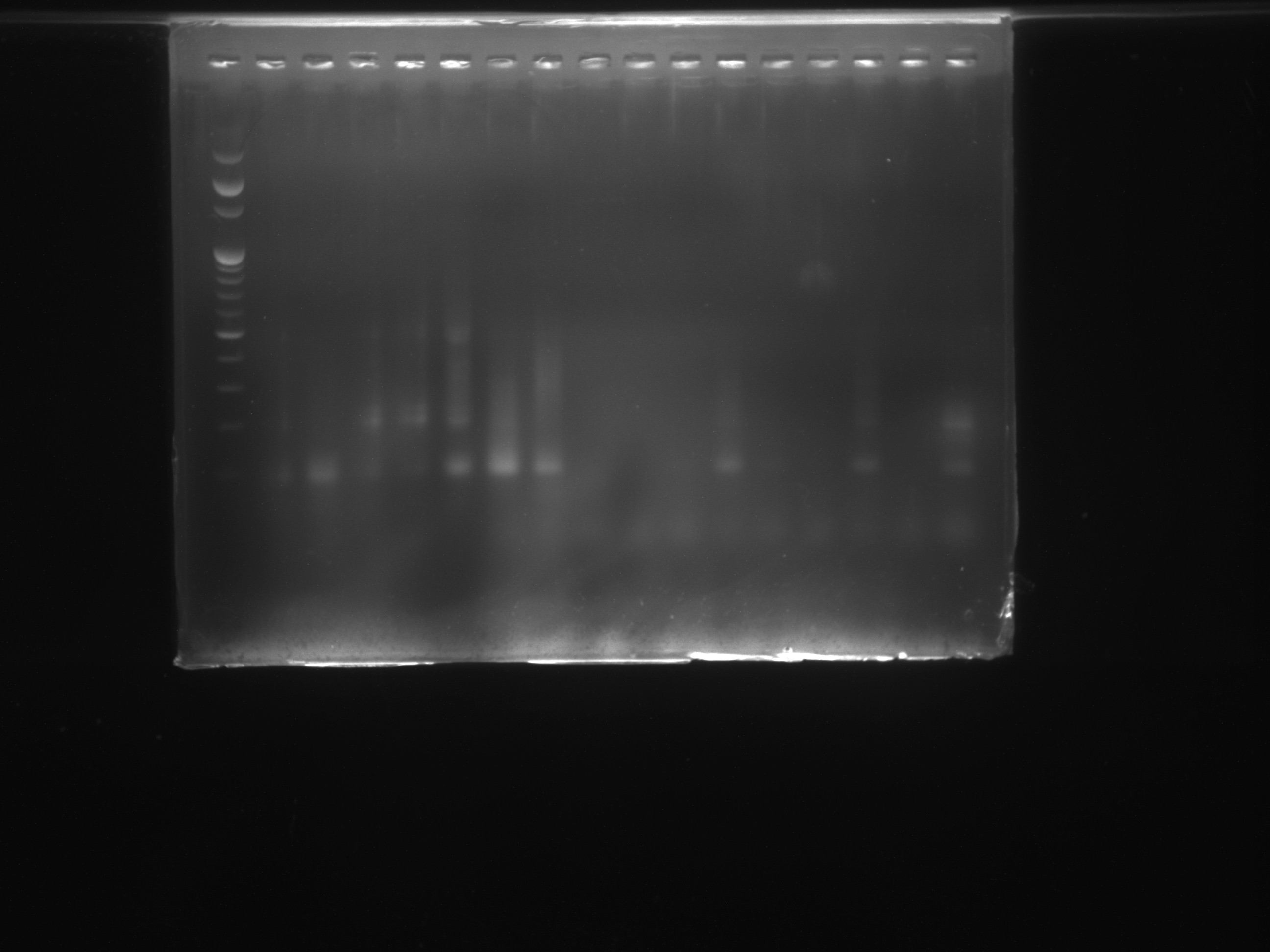

Supplement: Supplementary file 1 — Supplementary Material 1. [file 12870_2025_7148_MOESM1_ESM.zip › THR 3F (14~29) 1.jpg]

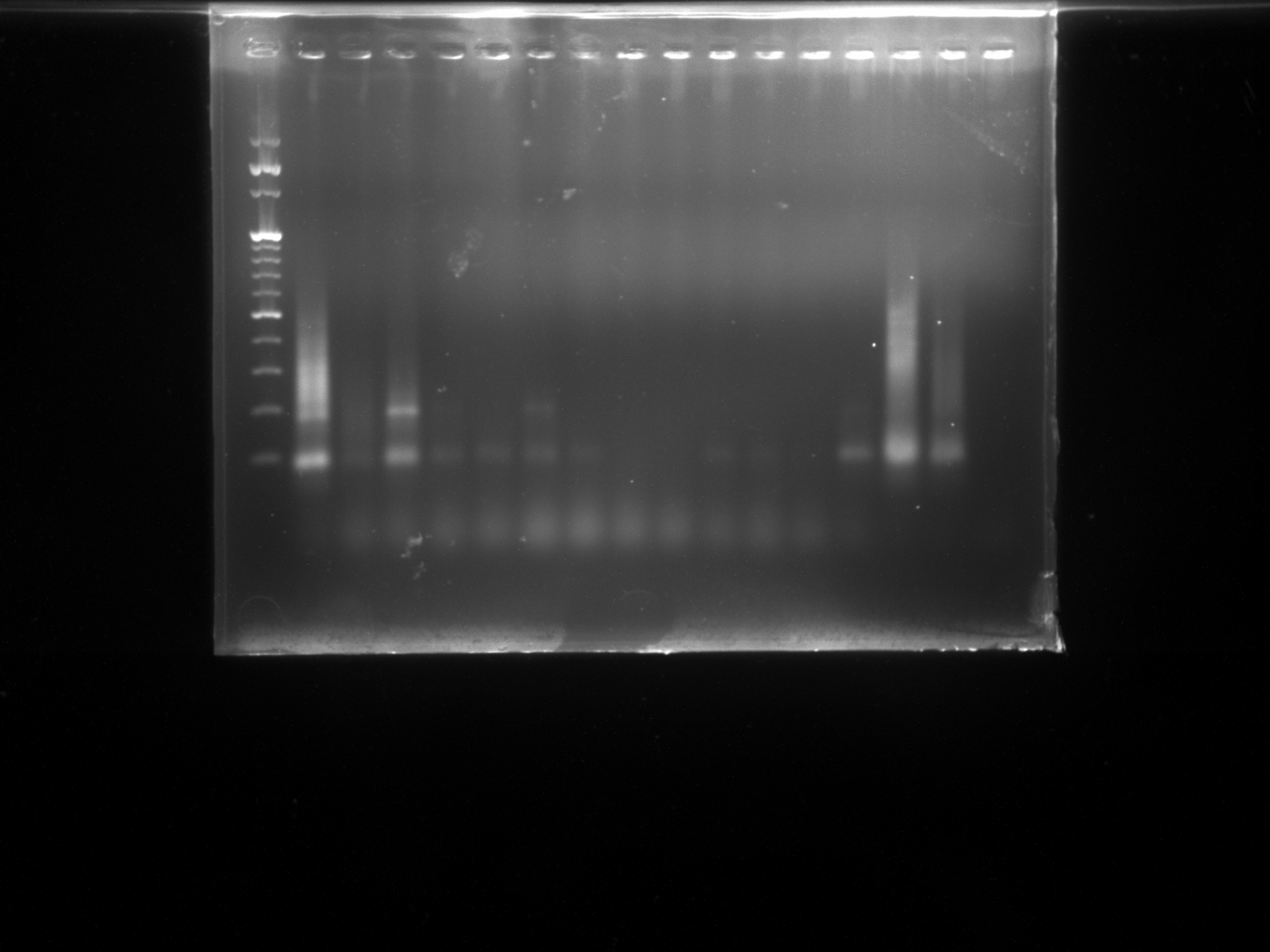

Supplement: Supplementary file 1 — Supplementary Material 1. [file 12870_2025_7148_MOESM1_ESM.zip › THR 3F (30~45) 1.jpg]

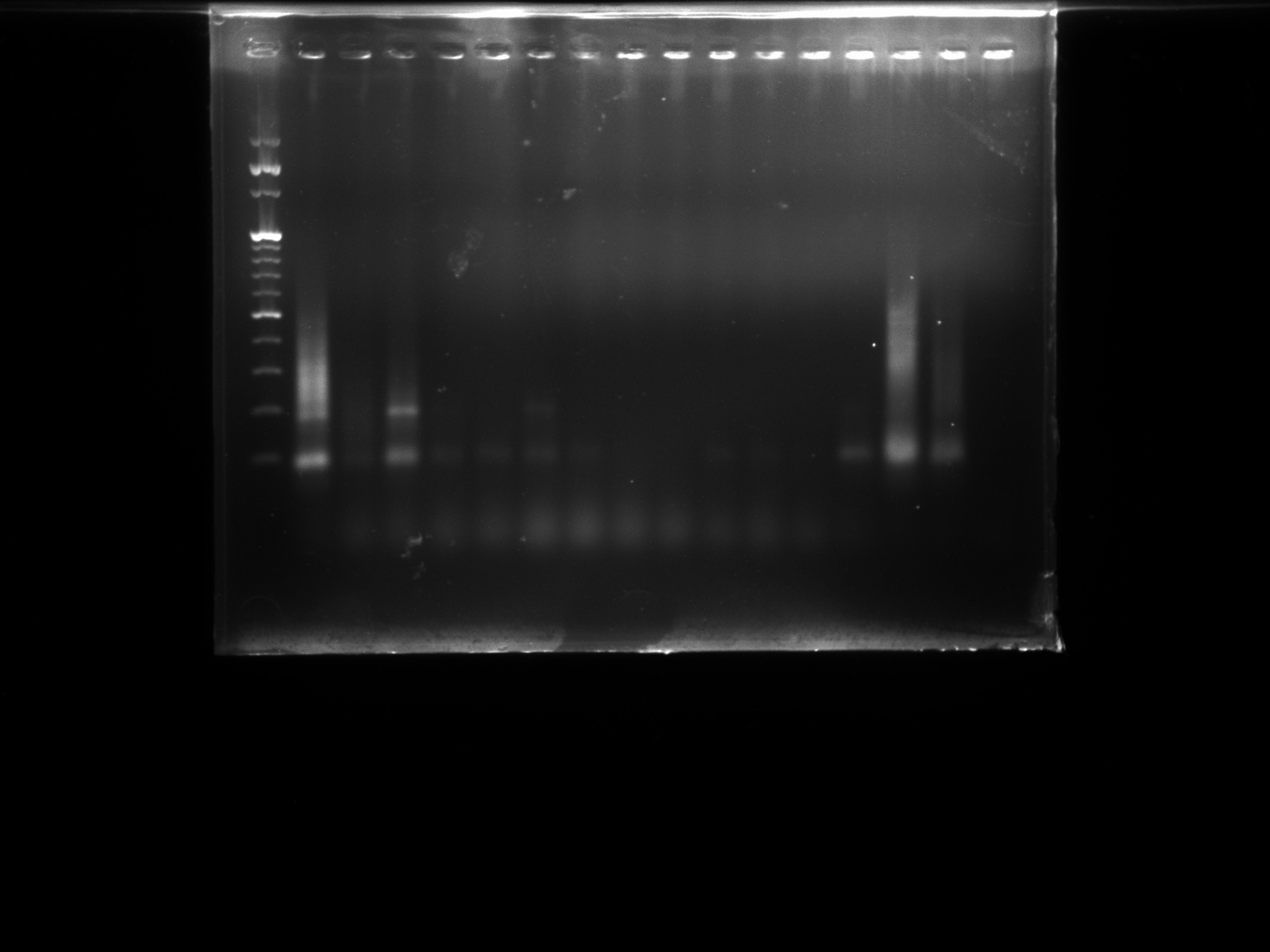

Supplement: Supplementary file 1 — Supplementary Material 1. [file 12870_2025_7148_MOESM1_ESM.zip › THR 3F (30~45) 2.jpg]

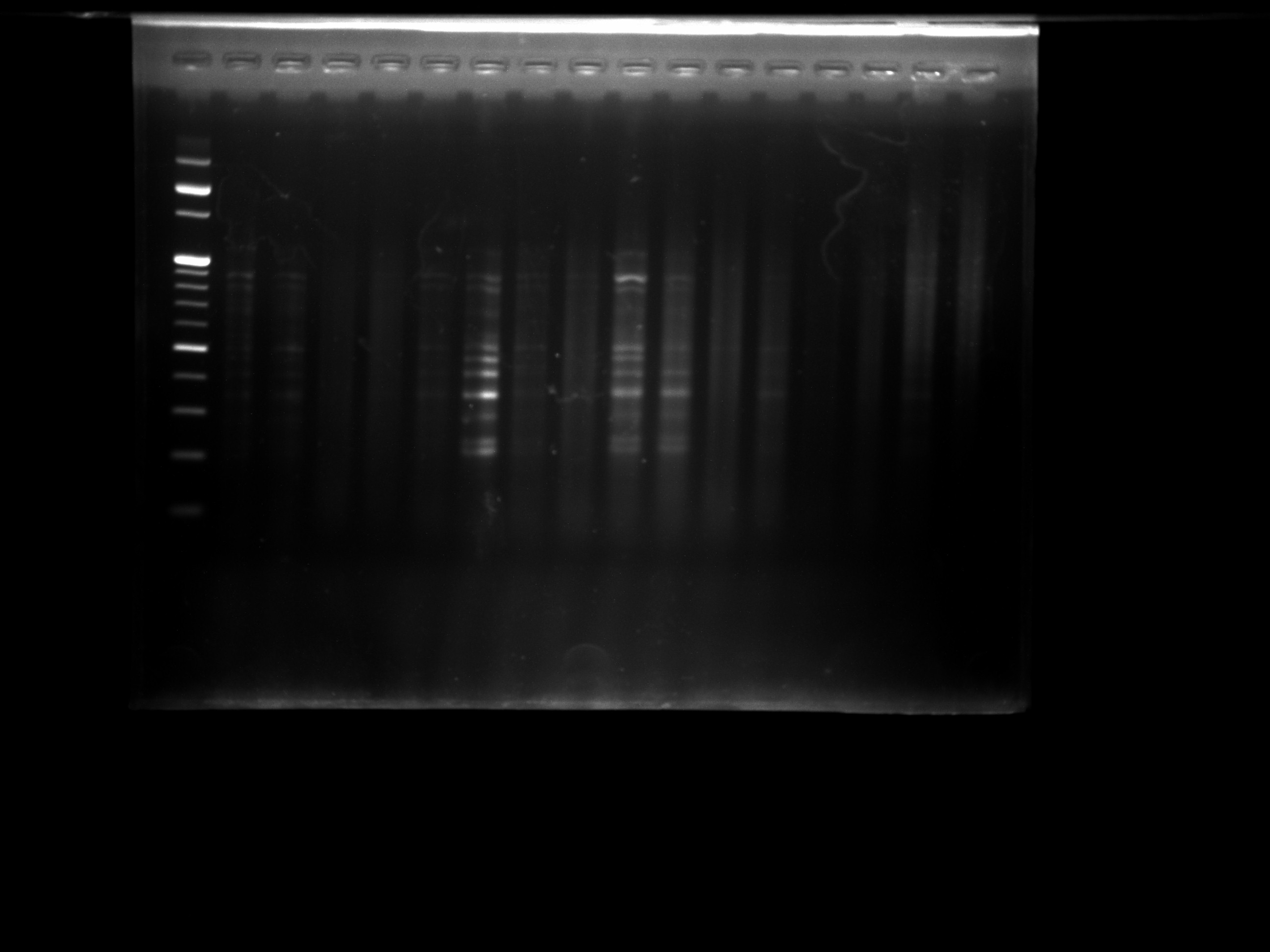

Supplement: Supplementary file 1 — Supplementary Material 1. [file 12870_2025_7148_MOESM1_ESM.zip › v1~13 polyphenol 1.jpg]

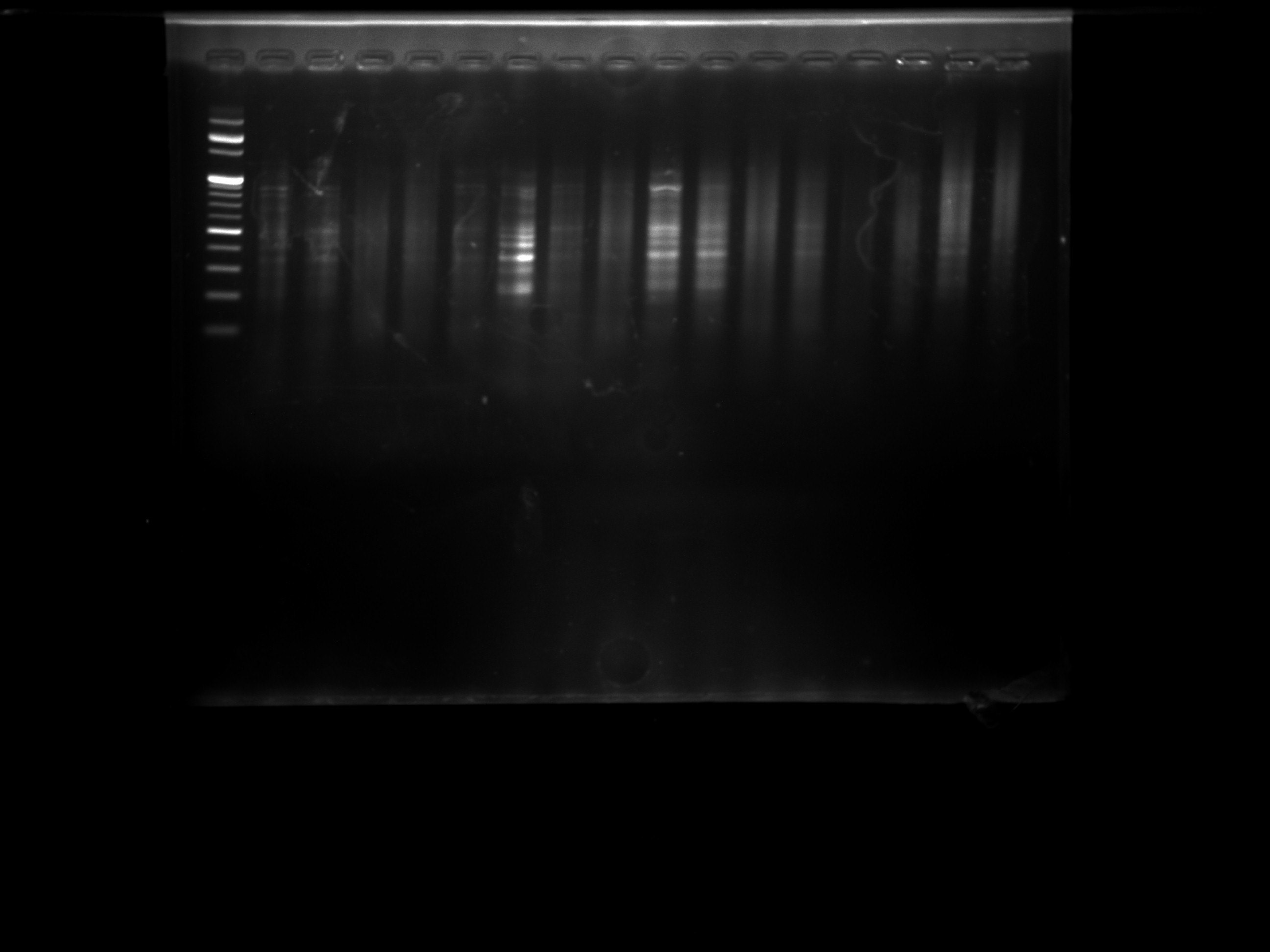

Supplement: Supplementary file 1 — Supplementary Material 1. [file 12870_2025_7148_MOESM1_ESM.zip › v1~13 polyphenol.jpg]
